# Supplementary material for: Polygenic score accuracy in ancient samples: Quantifying the effects of allelic turnover
Source: PLoS Genet. 2022 May 6;18(5):e1010170. doi: 10.1371/journal.pgen.1010170 (PMC9116686; doi:10.1371/journal.pgen.1010170)
Supplement: S1 Text — This supplementary text contains detailed derivations and additional analyses. (PDF) [file pgen.1010170.s001.pdf]

S1 Text. Extended model, methods, and results for "Polygenic score accuracy in ancient samples: quantifying the effects of allelic turnover"

Maryn O. Carlson[1*], Daniel P. Rice[2], Jeremy J. Berg[1,2], and Matthias Steinrücken[1,2,3*]

[1]Committee on Genetics, Genomics, & Systems Biology, University of Chicago, Chicago, IL, USA
[2]Department of Human Genetics, University of Chicago, Chicago, IL, USA
[3]Department of Ecology & Evolution, University of Chicago, Chicago, IL, USA
[*]To whom correspondence should be addressed: mocarlson@uchicago.edu, steinrue@uchicago.edu

April 14, 2022

Contents

1 Joint allele frequency density in the population split scenario **2**
2 Power to detect a significant association in a GWA study **2**
3 Simulation procedures **4**
4 Alternative prediction models **5**
4.1 Moments of the maximum-likelihood and threshold models 6
4.2 A comparison to the Best Linear Unbiased Predictor 7
5 Polygenic scores from centered and scaled GWAS data **9**
6 Spectral representation of the transition density **10**
7 Detailed derivations of the metrics **11**
7.1 A form of the polygenic score bias for arbitrary thresholds 12
7.2 Mean-squared error 15
7.3 Expected additive genetic variance 16
7.4 Approximate sample correlation coefficient 16
8 Expected accuracy in the UK Biobank **18**
9 Deriving approximations to the metrics **21**
10 Polygenic score bias for recent genic selection **25**
11 Fixation index and prediction accuracy **25**
12 Comparison to the results of Wang et al. 2020 **27**
13 Necessary moments, under neutrality and at stationarity **28**
References **30**

1 Joint allele frequency density in the population split scenario

In the main text, we claim that under our assumptions—namely, neutrality, constant population size, and stationarity—the modeling framework readily encompasses a simple population split scenario in which two populations diverged some τ_{split} generations ago and the ancient individual was sampled at τ (Fig 1B in the main text). Specifically, the split scenario is analogous to the single population scenario (Fig 1A in the main text) in which the ancient individual is sampled at $2\tau_{\text{split}} - \tau$. We derive the form of the joint allele frequency density for the demographic split scenario (Fig 1B in the main text) as proof.

For the scenario in Fig 1A in the main text, the joint allele frequency density at τ and the present is $f(z_\tau, z_0) = p(z_\tau, z_0; \tau)\kappa(z_\tau)$, where $\kappa(\cdot)$ is the stationary density,

$$\kappa(z) := \frac{1}{B(a,a)} z^{a-1}(1-z)^{b-1} := \frac{\pi(z)}{B(a,a)}. \tag{1}$$

For the split scenario, we must condition on the allele frequency at the time of the split. The joint density of a single site is then,

$$\begin{aligned} f(z_\tau, z_0) &= \int_{z_s=0}^{1} f(z_\tau, z_0 | Z(\tau_s) = z_s)\kappa(z_s) dz_s \\ &= \int_{z_s=0}^{1} p(z_s, z_\tau; \tau - \tau_{\text{split}}) p(z_s, z_0; \tau_{\text{split}})\kappa(z_s) dz_s, \end{aligned} \tag{2}$$

where z_s is the integration variable for the allele frequency at the time of the split. We can further simplify Equation 2 in S1 Text by twice substituting the spectral representation of the transition density (Section 6 in S1 Text),

$$\begin{aligned} &f(z_\tau, z_0) \\ &= \int_{z_s=0}^{1} \left(\sum_{k=0}^{\infty} \frac{e^{-\lambda_k(\tau_{\text{split}} - \tau)}}{\langle B_k, B_k \rangle_\pi} B_k(z_s) B_k(z_\tau)\pi(z_\tau) \right) \\ &\qquad \left(\sum_{m=0}^{\infty} \frac{e^{-\lambda_m \tau_{\text{split}}}}{\langle B_m, B_m \rangle_\pi} B_m(z_s) B_m(z_0)\pi(z_0) \right) \frac{\pi(z_s)}{B(a,b)} dz_s \\ &= \sum_{k=0}^{\infty} \frac{e^{-\lambda_k(2\tau_{\text{split}} - \tau)}}{\langle B_k, B_k \rangle_\pi} B_k(z_\tau) B_m(z_0) \frac{\pi(z_\tau)}{B(a,b)} \pi(z_0), \end{aligned} \tag{3}$$

where we exchanged integration and summation. We recognize that this equation is equal to,

$$f(z_\tau, z_0) = p(z_\tau, z_0; 2\tau_{\text{split}} - \tau) \frac{\pi(z_\tau)}{B(a,b)} = p(z_\tau, z_0; 2\tau_{\text{split}} - \tau)\kappa(z_s). \tag{4}$$

Thus, the joint density in the instance of a population split is of the same form as in the single population scenario, but with a modified time argument: $2\tau_{\text{split}} - \tau$ instead of τ.

2 Power to detect a significant association in a GWA study

We follow [1] (who follow [2]) in modeling the power of a GWA study to detect a significant association. We assume that conditional on the true effect size β_ℓ, and the population allele

frequency Z_ℓ (implicitly assuming $\hat{Z}_\ell \approx Z_\ell$) the estimated marginal effect $\hat{\beta}_\ell$ is normally distributed,

$$\hat{\beta}_\ell | \beta_\ell, Z_\ell \sim \mathcal{N}\left(\beta_\ell, \frac{V_p}{2nZ_\ell(1-Z_\ell)}\right), \tag{5}$$

where V_p is the total phenotypic variance which includes both genetic and environmental effects. Under the null hypothesis, i.e., $\beta_\ell = 0$, $\hat{\beta}_\ell$ is normally distributed with mean zero and the same variance as Equation 5 in S1 Text. The estimated contribution of locus ℓ to the phenotypic variance, $\hat{v}_\ell$, is $\hat{v}_\ell := 2\hat{\beta}_\ell^2 Z_\ell(1-Z_\ell)$. When normalized by $\frac{V_p}{n}$, $\hat{v}_\ell$ is chi-squared distributed with one degree of freedom,

$$\frac{\hat{v}_\ell}{V_p/n} = \frac{2\hat{\beta}_\ell^2 Z_\ell(1-Z_\ell)}{V_p/n} \sim \chi_1^2. \tag{6}$$

It follows that there is some threshold contribution to variance, v_*, such that the test statistic given in Equation 6 in S1 Text is statistically significant. Specifically, fixing the significance threshold α,

$$v_* = F^{-1}(1-\alpha) = 2\left(\text{erf}^{-1}(1-\alpha)\right)^2, \tag{7}$$

where $F^{-1}(\cdot)$ is the inverse cumulative distribution function (*cdf*) of a chi-squared distributed random variable with one degree of freedom, and erf is the error function (Equation A82 in [1]). This implies that a locus which satisfies,

$$\frac{2\hat{\beta}_\ell^2 Z_\ell(1-Z_\ell)}{V_p/n} > v_*, \tag{8}$$

will yield a statistically significant association. Equation 8 in S1 Text further implies that if, for a fixed Z_ℓ,

$$|\hat{\beta}_\ell| > \sqrt{\frac{v_*(V_p/n)}{2Z_\ell(1-Z_\ell)}}, \qquad \text{or, for a fixed } \hat{\beta}, \qquad Z_\ell(1-Z_\ell) > \frac{v_*(V_p/n)}{2\hat{\beta}_\ell^2}, \tag{9}$$

site ℓ will yield a significant association. If we substitute the true effect β_ℓ for $\hat{\beta}_\ell$ in Equation 9 in S1 Text, we can define these thresholds with respect to the true effect. And, for a fixed β our condition is,

$$\frac{1}{2} - \frac{1}{2}\sqrt{1 - \frac{2v_*(V_p/n)}{\beta_\ell^2}} < Z_\ell < \frac{1}{2} + \frac{1}{2}\sqrt{1 - \frac{2v_*(V_p/n)}{\beta_\ell^2}}. \tag{10}$$

We define,

$$\gamma_\ell = 1 - \sqrt{1 - \frac{2v_*(V_p/n)}{\beta_\ell^2}}, \qquad \text{and}, \qquad \beta_* = \sqrt{\frac{v_*(V_p/n)}{2Z_\ell(1-Z_\ell)}}. \tag{11}$$

Equation 11 in S1 Text specifies the threshold model given in Equation 3 in the main text, $D_\ell \in [d_\ell, 2n - d_\ell]$, where D_ℓ is the allele count in the GWA study and $d_\ell = \lceil n\gamma_\ell \rceil$. The distributional assumptions in Equation 5 in S1 Text imply that the threshold model will be a good approximation when n is large relative to V_p and the detection threshold is not too small. For example, if the allele frequency in the GWA study was at its minimum frequency of $\frac{1}{2n}$, then the variance of $\hat{\beta}$ would be proportional to V_p—which may be large.

3 Simulation procedures

In this section, we describe how we simulated the ancient polygenic scores (i) under neutrality and (ii) in the presence of genic selection.

Neutrality. To assess the accuracy of our theoretical results for the various statistics (introduced in **Analytical Results** in the main text), we simulated realizations of the polygenic score for ancient individuals according to our model (specified in **Model and metrics** in the main text).

Initialization. To initialize each realization, we sampled L population allele frequencies, $\boldsymbol{Z}(\tau) \in [0,1]^L$, from a Beta-distribution with parameters $a = 4N\mu$ and $b = 4N\nu$. As the population size N is finite, the beta-distribution is a continuous approximation to the discrete probability mass function governing the allele frequencies. Thus, we conduct one round of binomial sampling to obtain frequencies in the set $\{0, \frac{1}{2N}, \ldots, 1 - \frac{1}{2N}, 1\}$.

Allele frequency evolution. Allele frequencies then evolve forward-in-time until the present ($t = 0$) when the GWA study is conducted. For forward and backward mutation rates μ and ν, the transition probability of the discrete Wright-Fisher process is,

$$\psi_\mu(z) = (1-z)\mu + z(1-\nu) = \mu(1-2z) + z, \tag{12}$$

for an allele frequency $z \in [0,1]$, and where the second equality follows for $\mu = \nu$. Conditional on the allele frequency at t (generations in the past), the allele frequency in the subsequent generation is given by $(t-1)$,

$$Z_\ell(t-1)|Z_\ell(t) \sim Bin\left(2N, \psi_\mu\left(Z_\ell(t)\right)\right), \tag{13}$$

until $t - 1 = 0$.

Genome-wide association study. To conduct the GWA study, we sample n diploid genotypes, $\boldsymbol{X}_i(0) \in \{-1,0,1\}^L$, for $i \in \{1,\ldots,n\}$ conditional on the allele frequencies, $\boldsymbol{Z}(0)$. Conditional on $\boldsymbol{Z}(0)$, each genotype is *iid*, $\boldsymbol{X}_i(0)|\boldsymbol{Z}(0) \sim \prod_{\ell=1}^{L} Bin(2, Z_\ell(0))$. We then sample their phenotypes, $\boldsymbol{Y}(0) \in \mathbb{R}^n$ conditional on the their genotypes, according to Equation 1 in the main text. This set of n genotypes and phenotype comprise the GWA study sample.

To estimate the effects we first compute the allele count at each site D_ℓ in the study sample. If D_ℓ is within the specified interval $[d_\ell, 2n - d_\ell]$, we set the effect estimate to β_ℓ (Equation 3 in the main text). If D_ℓ falls outside of the interval, then the effect estimate is set to 0. We then estimate $\hat{C}$ using Equation 5 in the main text.

Sampling the ancient individual(s). We sample the genotype(s) $\boldsymbol{X}(\tau)$ and phenotype(s) $Y(\tau)$ of an ancient individual(s) conditional on the population allele frequencies $\boldsymbol{Z}(\tau)$.

Computing estimates of the statistics. We compute method of moments estimators for each of the statistics defined in *Quantifying out-of-sample prediction errors* in **Model and metrics** of the main text. For each ancient sampling time, $\tau = \{\tau_1, \tau_2, \ldots, \tau_T\}$, we conduct K simulations. For $bias(\tau)$, $mse(\tau)$, and $\hat{V}_A(\tau)$, we compute per-locus statistics, and thus average over all $L \times K$ independent locus trajectories for each time point. For example, the estimator of the *bias* is given by,

$$\overline{bias_\ell}(\tau) := \frac{1}{KL}\sum_{k=1}^{K}\sum_{\ell=1}^{L}\left(\bar{X}_{k\ell} - X_{k\ell}(\tau)\right)\left(\beta - \hat{\beta}_{k\ell}\right),$$

where k indexes the simulation and ℓ the locus. The estimator's $(1-\alpha)\%$ confidence interval is given by,

$$bias_\ell(\tau) \in [\overline{bias}_\ell(\tau) \pm z_{\alpha/2} s_{bias_\ell}]$$

where s_{bias_ℓ} is the estimated standard deviation of $bias_\ell(\tau)$ and $z_{\alpha/2}$ is the inverse cumulative distribution function of a standard normally distributed random variable evaluated at $\alpha/2$.

Our estimators for the sample correlation coefficient $r^2(\tau)$ and its approximation $\rho^2(\tau)$ are computed for each replicate of L loci. For example, for the k-th replicate,

$$r_k^2 := \frac{Cov[\hat{\boldsymbol{Y}}_k(\tau), \boldsymbol{Y}_k(\tau)]}{Var[\hat{\boldsymbol{Y}}_k(\tau)]Var[\boldsymbol{Y}_k(\tau)]} = \frac{Cov[\sum_{\ell=1}^L \boldsymbol{X}_{k\ell}(\tau)\hat{\beta}_{k\ell}, \sum_{\ell=1}^L \boldsymbol{X}_{k\ell}(\tau)\beta_{k\ell} + \boldsymbol{\epsilon}]}{Var[\sum_{\ell=1}^L \boldsymbol{X}_{k\ell}(\tau)\hat{\beta}_{k\ell}]Var[\sum_{\ell=1}^L \boldsymbol{X}_{k\ell}(\tau)\beta_{k\ell} + \boldsymbol{\epsilon}]}, \tag{14}$$

where $\hat{\boldsymbol{Y}}_k(\tau)$ and $\boldsymbol{Y}_k(\tau)$ are the n_a-length vectors of ancient polygenic scores and phenotypes, respectively; $\boldsymbol{X}_{k\ell}(\tau)$ is the vector of ancient genotypes at the ℓ-th site; and $\boldsymbol{\epsilon}$ is the vector of environmental contributions to each individual's phenotype. Our estimator $\hat{r}^2$ is an average of the K realizations of r_k^2. To estimate $\rho^2(\tau)$, we first find estimators for the covariance and variance terms in Equation 14 in S1 Text by averaging over the K simulations. We then compute the ratio of these quantities to compute $\hat{\rho}^2(\tau)$.

Genic selection. To investigate how positive selection influences the statistical properties of polygenic scores, we simulated recent directional selection. The population evolves neutrally until the onset of selection τ_s years in the past. The A_2 allele confers a fitness advantage of s, such that the relative fitnesses of the genotypes A_1A_1:A_1A_2:A_2A_2 are given by 1:$(1+s)$:$(1+2s)$.

Initialization. To initialize each realization, we sample L population allele frequencies, $\boldsymbol{Z}(\tau) \in [0,1]^L$, from the stationary distribution of the neutral Wright-Fisher diffusion with recurrent mutation. If the ancient sampling time τ is more recent than τ_s, we simulate 50 generations of neutral evolution before the onset of selection.

Allele frequency evolution. Allele frequencies evolve neutrally until the onset of selection at τ_s. At this juncture, the allele frequencies begin to evolve according to,

$$\psi_{\mu s}(z) = \frac{\left[(1-z)^2 + z(1-z)(1+s)\right]\mu + \left[z(1-z)(1+s) + z^2(1+2s)\right](1-\mu)}{\bar{w}(z)}, \tag{15}$$

where μ is the forward and backward per-locus, per-generation mutation rate, and the denominator is the mean fitness in a population with A_2 allele frequency z, up to the present day. The simulations with selection are otherwise identical to those under neutrality.

4 Alternative prediction models

In the main text, we introduced a simple threshold model for the effect estimates (Equation 3 in the main text). Here, we first consider a more realistic model in which the effect estimate $\hat{\beta}_\ell$ is the maximum-likelihood estimate (MLE) of β_ℓ (in Section 4.1 in S1 Text). We give expressions for the first two moments of $\hat{\beta}_\ell$ conditional on the contemporary allele frequencies Z_ℓ under each model. In doing so, we illustrate the additional challenges posed by the MLE model, and why we ultimately opted to pursue the simpler threshold model. We next relate the threshold model to the best linear unbiased predictor, or BLUP (e.g., [3,4]; Section 4.2 in S1 Text). In doing so, we provide a brief exposition of BLUP following [4].

4.1 Moments of the maximum-likelihood and threshold models

Maximum-likelihood threshold model. We define the MLE threshold model,

$$\hat{\beta}_\ell := \begin{cases} \frac{Cov[\boldsymbol{X}_\ell, \boldsymbol{Y}]}{Var[\boldsymbol{X}]} = \frac{\sum_{i=1}^{n}(Y_i - \bar{Y})(X_{i\ell} - \bar{X}_\ell)}{\sum_{i=1}^{n}(X_{i\ell} - \bar{X}_\ell)^2} & \text{if } D_\ell \in [d_\ell, 2n - d_\ell] \\ 0 & \text{else,} \end{cases} \tag{16}$$

where each genotype, phenotype pair $(\boldsymbol{X}_i, Y_i)$ for $i \in \{1, \ldots, n\}$ is associated with an individual in the GWA study; $Cov[\cdot, \cdot]$ and $Var[\cdot]$ are the sample covariance and variance, respectively; and, as before, $\bar{X}_\ell$ and $\bar{Y}$ are the average genotype at the ℓ-th locus and phenotype in the GWA sample.

First moment of $\hat{\beta}$. For both models, it can be shown that,

$$\mathbb{E}\left[\hat{\beta}_\ell | Z_\ell, \beta_\ell\right] = \beta_\ell \wp(Z_\ell, 2n, d_\ell), \tag{17}$$

where Z_ℓ is the contemporary population allele frequency; and

$$\wp(z_\ell, 2n, d_\ell) = \sum_{i=d_\ell}^{2n-d_\ell} \binom{2n}{i} z_\ell^i (1 - z_\ell)^{2n-i} \tag{18}$$

is the probability that the allele count in the GWA study falls at or above the threshold d_ℓ. Thus, when the site is segregating at a sufficiently high frequency in the GWA study sample, the estimator is unbiased. Unconditionally, for an allele count threshold d_ℓ, $\mathbb{E}[\hat{\beta}_\ell | \beta_\ell] = \beta_\ell(1 - 2P^{(d_\ell)})$, where $P^{(d_\ell)}$ is the *cdf* of a beta-binomial random variable and is defined in Equation 42 in S1 Text.

Second moment of $\hat{\beta}$. The two models yield different second moments.

Simple threshold model. It can be shown that under the simpler model,

$$\mathbb{E}\left[\hat{\beta}_\ell^2 | \boldsymbol{Z}, \beta_\ell\right] = \beta_\ell^2 \wp(Z_\ell, 2n, d_\ell). \tag{19}$$

As Equation 19 in S1 Text only involves the allele frequency of site ℓ, it is not influenced by variation at other sites in $\mathcal{L}$. And, unconditionally, $\mathbb{E}[\hat{\beta}_\ell^2 | \beta_\ell] = \beta_\ell^2 \left(1 - 2P^{(d_\ell)}\right)$.

MLE model. It can be shown that under the MLE model,

$$\mathbb{E}\left[\hat{\beta}_\ell^2 | \boldsymbol{Z}(0)\right] \approx \left(\beta_\ell^2 + \sum_{\ell' \neq \ell} \beta_{\ell'}^2 \frac{Z_{\ell'}(1 - Z_{\ell'})}{nZ_\ell(1 - Z_\ell)} + \frac{\sigma_e^2}{2nZ_\ell(1 - Z_\ell)}\right) \wp(Z_\ell, 2n, d_\ell), \tag{20}$$

where the sum is over all loci $\ell' \in \mathcal{L}$ such that $\ell' \neq \ell$. The approximation is due to approximating the expectation of a ratio with the ratio of expectations, and as such, comes with all of the corresponding dangers of such an approximation. In addition, we can see from Equation 20 in S1 Text that the second moment of $\hat{\beta}_\ell$ depends on the allele frequencies at all other loci in $\mathcal{L}$. While were able to compute the metrics under this approximate MLE model, we concluded that its reliance on strict assumptions about the genetic architecture (via the second moment) obscured the effects of allelic turnover. In addition, a threshold model arises naturally as the large n limit of the MLE model which is, up to a sample size factor, equivalent to Equation 5 in S1 Text when the allele frequency Z_ℓ is not too small.

4.2 A comparison to the Best Linear Unbiased Predictor

A brief overview of BLUP. We follow the Supporting Information of de los Campos et al. [4] in introducing BLUP. However, we use notation consistent with the notation of our investigation.

The model underlying BLUP is of the form,

$$\tilde{\boldsymbol{Y}} = \tilde{\boldsymbol{X}}\boldsymbol{\beta} + \epsilon, \tag{21}$$

where for a GWA study consisting of n individuals, $\tilde{\boldsymbol{Y}} \in \mathbb{R}^n$ is a vector of centered and scaled phenotypes, $\boldsymbol{\beta} \in \mathbb{R}^{L'}$ is a vector of marker effects, and $\tilde{\boldsymbol{X}} \in \mathbb{R}^{n \times L'}$ is a centered and scaled matrix of the genotypes at all L' *segregating* sites in the genome (potentially above some minimum allele frequency), with the $i\ell$-th element $\tilde{X}_{i\ell} = \frac{X_{i\ell} - \bar{X}_\ell}{\sqrt{2\hat{Z}_\ell(1-\hat{Z}_\ell)}}$, where $X_{i\ell} \in \{-1, 0, 1\}$ is the individual's genotype, and $\bar{X}_\ell$ and $\hat{Z}_\ell$ are the average genotype and estimated allele frequency in the GWA study sample. (Note that centering and scaling of the genotypes and phenotypes is not necessary to the formulation of BLUP [4].) The marker effects are assumed *iid*, $\beta_\ell \sim \mathcal{N}(0, \sigma_\beta^2)$, where σ_β^2 is the prior variance of the marker effects. Similarly, the residuals are assumed *iid* with $\epsilon_i \sim \mathcal{N}(0, \sigma_e^2)$, where σ_e^2 is the prior residual variance. Under this model, the effect estimates and phenotypes follow a multivariate normal distribution,

$$\begin{bmatrix} \boldsymbol{\beta}|\tilde{\boldsymbol{X}} \\ \tilde{\boldsymbol{Y}}|\tilde{\boldsymbol{X}} \end{bmatrix} \sim MVN\left(0, \begin{bmatrix} \boldsymbol{I}_{L'}\sigma_\beta^2 & \tilde{\boldsymbol{X}}^T\sigma_\beta^2 \\ \tilde{\boldsymbol{X}}\sigma_\beta^2 & \tilde{\boldsymbol{X}}\tilde{\boldsymbol{X}}^{\boldsymbol{T}}\sigma_\beta^2 + \boldsymbol{I}_n\sigma_e^2 \end{bmatrix}\right), \tag{22}$$

such that,

$$\mathbb{E}[\hat{\boldsymbol{\beta}}|\tilde{\boldsymbol{Y}}, \tilde{\boldsymbol{X}}] = (1/L')\tilde{\boldsymbol{X}}^T[\boldsymbol{G} + \boldsymbol{I}_n(\sigma_\epsilon^2/(L'\sigma_\beta^2)]\tilde{\boldsymbol{Y}} \tag{23}$$

with $\boldsymbol{G} = (1/L')\tilde{\boldsymbol{X}}\tilde{\boldsymbol{X}}^T$. And thus,

$$\mathbb{E}[\hat{\beta}_\ell|\tilde{\boldsymbol{Y}}, \tilde{\boldsymbol{X}}] = (1/L')\tilde{\boldsymbol{X}}_{\boldsymbol{\ell}}^T[\boldsymbol{G} + \boldsymbol{I}_n(\sigma_\epsilon^2/(L'\sigma_\beta^2)]\tilde{\boldsymbol{Y}}. \tag{24}$$

Relating the MLE estimate. When we compare Equation 24 in S1 Text to the MLE estimate (for centered and scaled genotypes and phenotypes), i.e.,

$$\hat{\beta}_\ell = \tilde{X}_\ell^T\tilde{\boldsymbol{Y}}, \tag{25}$$

we see that they are analogous up to the bracketed expression in Equation 24 in S1 Text. This term, $\boldsymbol{G} + \boldsymbol{I}_n(\sigma_\epsilon^2/L'\sigma_\beta^2)$, models the relationship between individuals in the GWA study and affects the effect estimates of all loci. If the relationship matrix $\boldsymbol{G}$ were the identity, than the bracketed term would simply scale the effect estimates by a factor $\sigma_\epsilon^2/\sigma_\beta^2$. However, in the likely instance that $\boldsymbol{G}$ deviates from the identity, the primary contribution to the per-locus effect estimate is still given by the covariance between the genotype of locus ℓ and the phenotype, i.e., by Equation 25 in S1 Text. Thus, the MLE of the effect size is closely related to the effect estimate derived from BLUP, at least when the variance components are known.

A dense trait architecture. When the trait architecture is dense, a large number of segregating sites each impart a small effect on the trait (see [5] for the limiting behavior, referred to as the infinitesimal model). In this setting, BLUP—which allows all loci to have non-zero effects—will likely yield predictions with higher accuracy than the "prune and threshold" model—which assumes only one "causal" site within a given genomic window. As noted in the *Discussion*, our assumption of linkage equilibrium between loci necessarily breaks down under a dense architecture, and the allele frequency trajectories cannot be modeled independently. (As before, we assume that all loci

are evolving neutrally in a constant size population.) In addition, the marginal effect estimate for a given locus will absorb the effects of its neighbors in proportion to the LD between these loci (e.g., see Equation A87 of [1]). Genome-wide association study and prediction methods that model LD between loci, e.g. [6], aim to reduce to the effects of the latter.

Let d be the allele frequency threshold at which point a locus is considered "segregating" in the GWA study, e.g. $d = 1$. (Though, standard quality control pipelines may impose a threshold $d > 1$ to remove very low frequency sites that are more susceptible to false positives.) The above two LD-induced complications aside, we can loosely approximate the BLUP model by using d as the allele frequency threshold *for all loci* irrespective of their effect sizes. Accuracy under the BLUP can then be roughly approximated as,

$$\begin{aligned}\rho^2(\tau) &\approx \frac{\frac{1}{2a+1}\sum_{\ell'=1}^{L'}\beta_\ell^2[a(1-2P^{(d)})+2e^{-(2a+1)\tau}P_3^{(d)}]}{\frac{a}{2a+1}\sum_{\ell'=1}^{L'}\beta_\ell^2+\sigma_e^2} \\ &= \frac{\frac{1}{2a+1}[a(1-2P^{(d)})+2e^{-(2a+1)\tau}P_3^{(d)}]}{\frac{a}{2a+1}+\sigma_{e''}^2},\end{aligned} \tag{26}$$

where $\sigma_{e''}^2 = \sigma_e^2/(\sum_{\ell'}\beta_{\ell'}^2)$, and the sum is over all segregating sites $\ell' \in \{1,\dots,L'\}$. As the threshold d does not depend on the per-locus effect, we can factor the sum of the squared effect sizes to arrive at the second line of Equation 26 in S1 Text. Relative accuracy readily follows from Equation 26 in S1 Text,

$$\rho^2(\tau)/\rho^2(0) \approx \frac{a(1-2P^{(d)})+2e^{-(2a+1)\tau}P_3^{(d)}}{a(1-2P^{(d)})+2P_3^{(d)}}. \tag{27}$$

We can compare these expressions with those derived in Section 7.4 in S1 Text. (i) As before, relative accuracy does not depend on the effect sizes, and will cohere with our previous results for $d_{\ell'} = d$ for all ℓ'. When the mutation rate is small, most sites are fixed for either the A_1 or A_2 allele. Thus, many sites will evade detection even when $d \leq 10$, and both accuracy and relative accuracy will decay substantially over time. In addition, relative accuracy appears insensitive to the threshold d (see insets in Fig 3 in the main text), thus relative accuracy, given in Equation 27 in S1 Text, will likely behave similarly under BLUP. And (ii), while Equation 26 in S1 Text removes the relationship between an effect size $\beta_{\ell'}$ and its threshold $d_{\ell'}$, we still expect it to behave qualitatively similar to our previous results. Though, as $d \leq d_{\ell'}$ for all ℓ' under our previous parameterization (see Section 2 in S1 Text), BLUP will achieve higher accuracy than the threshold model.

Simply setting $d_{\ell'} = d = 1$ for all $\ell' \in \{1,\dots,L'\}$ to model BLUP does not, however, capture an important way in which BLUP deviates from the "prune and threshold" model. While not captured explicitly in our threshold model, the standard error of the effect estimate $\hat{\beta}_{\ell'}$ for locus ℓ' depends on both the magnitude of its true effect and the allele frequency. In particular, all else being equal, a variant at low frequency will have a larger standard error (and thus larger p-value) compared to a variant at moderate frequency with the same effect. We justify ignoring noise in the effect estimates in our modeling of the "prune and threshold" approach as variants exceeding the p-value threshold must either be at intermediate frequency and/or of large effect. Thus, this effect should be mitigated by the fact that standard errors of loci with non-zero effects in the polygenic score are necessarily small relative to the estimated effect size.

In contrast, BLUP allows *all* variants to have non-zero effects, implying that low frequency variants will have systematically larger standard errors relative to moderate frequency variants. As low frequency variants shift towards higher frequencies in the ancient population, these variants will disproportionately—relative to their frequencies in the ancient population—contribute to the

noisiness of BLUP. Similarly, as moderate frequency variants shift towards lower frequencies in the ancient population, they will contribute disproportionately less to the prediction noise. Future work may precisely quantify the confluence of allelic turnover and effect estimate uncertainty induced by the BLUP model (and perhaps under the "prune and threshold" model as well). In addition, a more rigorous analysis would necessarily take into account LD between loci.

5 Polygenic scores from centered and scaled GWAS data

In the main text, we chose not to center and scale the genotypes and phenotypes of sampled individuals when conducting the GWA study. In this section, we show that our conclusions are robust to this choice. Our calculations also demonstrate that procedures convenient for statistical analysis, namely scaling, prove inconvenient when evolutionary processes are taken into account.

Centering and scaling. We center and scale to unit variance the phenotypes and genotypes in the GWA study,

$$\tilde{Y}_i = \frac{Y_i - \bar{Y}}{s_Y} \qquad \text{and} \qquad \tilde{X}_{i\ell} = \frac{X_{i\ell} - \bar{X}_\ell}{s_\ell}, \tag{28}$$

where s_Y and s_ℓ are the sample standard deviations of the phenotypes and genotypes at locus ℓ, respectively. In this case, the marginal effect estimate of locus ℓ will be,

$$\tilde{\beta}_\ell = \frac{Cov[\tilde{\boldsymbol{X}}_\ell, \tilde{\boldsymbol{Y}}]}{Var[\tilde{\boldsymbol{X}}_\ell]} = \frac{1}{n}\sum_{i=1}^{n}(\tilde{X}_{i\ell} - 0)(\tilde{Y}_i - 0) = \frac{1}{ns_\ell s_Y}\sum_{i=1}^{n}(X_{i\ell} - \bar{X}_\ell)(Y_i - \bar{Y}) = \frac{s_\ell \hat{\beta}_\ell}{s_Y}. \tag{29}$$

Thus, with normalized genotypes and phenotypes, the effect estimate is scaled by a factor $\frac{s_\ell}{s_Y}$, but is otherwise unaltered.

The polygenic score in the transformed case $\hat{Y}^*$, ignoring any intercept term (which would be 0), is,

$$\hat{Y}_i^* = \sum_{\ell=1}^{L} \tilde{\beta}_\ell \tilde{X}_{i\ell} = \frac{1}{s_Y}\sum_{\ell=1}^{L} \hat{\beta}_\ell (X_{i\ell} - \bar{X}_\ell). \tag{30}$$

With centering and scaling, the polygenic score is a genetic prediction less the average genetic prediction in the GWA study sample, both scaled by a factor of s_Y.

Bias. We can compute the *bias* of the rescaled polygenic score as,

$$\begin{aligned}
\mathbb{E}\left[s_Y \hat{Y}_i^* - (Y_i - \bar{Y})\right] &= \sum_{\ell=1}^{L} \mathbb{E}\left[\hat{\beta}_\ell X_{i\ell}\right] - \sum_{\ell=1}^{L} \mathbb{E}\left[\hat{\beta}_\ell \bar{X}_\ell\right] - \mu - \sum_{\ell=1}^{L} \left[\beta_\ell X_{i\ell}\right] + \mathbb{E}\left[\bar{Y}\right] \\
&= \sum_{\ell=1}^{L} \mathbb{E}\left[\left(\hat{\beta}_\ell - \beta_\ell\right) X_{i\ell}\right] + \sum_{\ell=1}^{L} \mathbb{E}\left[\hat{\beta}_\ell \bar{X}_\ell\right] - \mu + \mu + \sum_{\ell=1}^{L} \beta_\ell \bar{X}_\ell \\
&= \sum_{\ell=1}^{L} \mathbb{E}\left[\left(\hat{\beta}_\ell - \beta_\ell\right)\left(X_{i\ell} - \bar{X}_\ell\right)\right].
\end{aligned} \tag{31}$$

Equation 31 in S1 Text shows that when we center and scale the data, we arrive at the same result. (One must rescale either $\tilde{Y}_i$ or $Y_i - \bar{Y}$ by s_Y or its inverse, respectively, to put the polygenic score and the phenotype on the same scale. The former is more mathematically convenient.)

Mean-squared error. The proof for the *mse* is almost identical to that for the *bias*. We thereby omit it.

Additive genetic variance. If the effect estimates $\tilde{\boldsymbol{\beta}}$ are used instead of $\hat{\boldsymbol{\beta}}$, one must rescale the estimate of heterozygosity from the ancient sample by that estimated in the GWA study (see Equation 1 of Supplementary Note 1 of Wang et al. [7] for a related procedure),

$$\tilde{V}_A(\tau) = 2\sum_{\ell=1}^{L} \mathbb{E}\left[\tilde{\beta}_\ell^2 \left(\frac{1}{s_\ell^2}\right) \hat{Z}_\ell(\tau)\left(1 - \hat{Z}_\ell(\tau)\right)\right] = 2\sum_{\ell=1}^{L} \mathbb{E}\left[\left(\frac{1}{s_Y}\right)^2 \hat{\beta}_\ell^2 \hat{Z}_\ell(\tau)\left(1 - \hat{Z}_\ell(\tau)\right)\right]. \quad (32)$$

This formulation is less convenient because it has random quantities in both the numerator and the denominator. Given that the units in which Y is measured are arbitrary, we forewent coping with the additional complexity imposed by this scaling.

Correlation coefficient. By similar arguments, one can show that the sample correlation coefficient is not influenced by centering and scaling.

6 Spectral representation of the transition density

Because the spectral representation of the transition density of an allele frequency is so central to our work, we provide a concise exposition here. For a lengthier treatment, we refer the reader to [8] and [9].

We represent the Wright-Fisher diffusion by its backward generator, $\mathscr{L}$. Introducing the quantities $a = 4N\mu$ and $b = 4N\nu$ for the population scaled mutation rates, $\mathscr{L}$ is given by,

$$\mathscr{L}f(z) = \frac{1}{2}z(1-z)\frac{\partial^2}{\partial z^2}\{f(z)\} + \frac{1}{2}[a(1-z) - bz]\frac{\partial}{\partial z}\{f(z)\}, \quad (33)$$

where z is frequency of the A_2 allele, and f is a twice continuously differentiable bounded function on $[0, 1]$ [8].

The transition density of the Wright-Fisher diffusion, $p(z, z'; t)$, specifies the likelihood of transitioning from allele frequency z to z' in a time interval $[t, 0]$. The spectral representation expresses the transition density as an infinite sum,

$$p(z, z'; t) = \sum_{j=0}^{\infty} c_j(z')e^{-\lambda_j t}R_j(z), \quad (34)$$

where, for $j = 0, 1, 2, \ldots$, $c_j(\cdot)$ is a constant factor that depends on the initial condition, defined below in Equation 36 in S1 Text; λ_j is the eigenvalue that corresponds to eigenfunction $R_j(\cdot)$; and $R_j(\cdot)$ is the j-th eigenfunction. The function, $\pi(\cdot)$, is the stationary measure and $\langle\cdot,\cdot\rangle_\pi$ is the inner product with respect to this measure, defined in Equation 37 in S1 Text. In the neutral, recurrent mutation model, the stationary measure $\pi(\cdot)$ is given by,

$$\pi(z) = z^{a-1}(1-z)^{b-1}, \quad (35)$$

where a and b are the population-scaled mutation rates defined in Equation 33 in S1 Text. Notice that Equation 35 in S1 Text is equivalent to the unnormalized density of a beta-distributed random variable with shape parameters a and b. When normalized to integrate to one, $\pi(\cdot)$ is the stationary density $\kappa(\cdot)$, first defined in Equation 1 in S1 Text. When the initial condition is a point mass at the initial allele frequency, z, the factor $c_j(z')$ is,

$$c_j(z') = \frac{R_j(z')\pi(z')}{\langle R_j, R_j\rangle_\pi}, \quad (36)$$

where $\langle R_j, R_j\rangle_\pi$ is the inner product of $R_j(\cdot)$ with itself. We also refer to an inner product of the form $\langle R_j, R_j\rangle_\pi$ as the squared norm of the j-th eigenfunction. More generally, we define the inner product of two arbitrary functions, $f(\cdot)$ and $g(\cdot)$ with respect to the stationary measure as,

$$\langle f, g\rangle_\pi := \int_{y=0}^{1} f(y)g(y)\pi(y)dy. \tag{37}$$

The inner product of two eigenfunctions, R_j and R_k, is then a special case of Equation 37 in S1 Text, with

$$\langle R_j, R_k\rangle_\pi = \begin{cases} \Delta_j(a,b) & \text{for } k = j, \\ 0 & \text{else,} \end{cases} \tag{38}$$

where,

$$\Delta_j(a,b) = \frac{\Gamma(j+a)\Gamma(j+b)}{(2j+a+b-1)\Gamma(j+a+b-1)\Gamma(j+1)}, \tag{39}$$

and $\Gamma(z) = \int_0^\infty x^{z-1}e^{-x}dx$ for $z \in \mathbb{R}$. Our work involves many inner products of the form, $\langle R_j, P_k\rangle_\pi$, where $P_k(\cdot)$ is a polynomial of degree k.

In the neutral recurrent mutation model, the eigenfunctions of the Wright-Fisher diffusion are Jacobi polynomials [8]. The Jacobi polynomials are polynomials of increasing order coincident with their indices, $j = 0, 1, 2, \ldots$, and obey a three-term recurrence relation,

$$\begin{aligned} zR_j(z) = &\frac{(j+a-1)(j+b-1)}{(2j+a+b-1)(2j+a+b-2)} R_{j-1}(z) \\ &+ \left[\frac{1}{2} - \frac{b^2 - a^2 - 2(b-a)}{2(2j+a+b)(2j+a+b-2)}\right] R_j(z) \\ &+ \frac{(j+1)(j+a+b-1)}{(2j+a+b)(2j+a+b-1)} R_{j+1}(z). \end{aligned} \tag{40}$$

For $j = 0$,

$$zR_0(z) = \frac{a}{a+b}R_0(z) + \frac{1}{a+b}R_1(z), \tag{41}$$

with $R_0(z) \equiv 1$.

In our work we will exploit two properties of the Jacobi polynomials: (i) the orthogonality of the eigenfunctions, i.e., Equation 38 in S1 Text, and (ii) the fact that a Jacobi polynomial of degree j is orthogonal to all lower order polynomials (of degree k, $k < j$).

7 Detailed derivations of the metrics

We provide detailed derivations of the metrics used to characterize ancient polygenic scores. For all but $bias(\tau)$, we restrict ourselves to equal detection thresholds, although our framework readily accommodates asymmetric detection thresholds. In order to represent the metrics succinctly, we

introduce several variables:

$$\begin{aligned}
P^{(d)} &:= \sum_{i=0}^{d-1} \binom{2n}{i} \frac{B(a+i, a+2n-i)}{B(a,a)} \\
P_1^{(d)} &:= \sum_{i=0}^{d-1} \left(\frac{i-n}{n}\right)^2 \binom{2n}{i} \frac{B(a+i, a+2n-i)}{B(a,a)} \\
P_2^{(d)} &:= \sum_{i=0}^{d-1} \left(\frac{(i-n)^2}{n(a+n)}\right) \binom{2n}{i} \frac{B(a+i, a+2n-i)}{B(a,a)} = \left(\frac{n}{a+n}\right) P_1^d \\
P_3^{(d)} &:= \sum_{i=0}^{d-1} \binom{2n}{i} \frac{B(a+i, a+2n-i)}{B(a,a)} \left(\frac{(2a+1)i(i-2n)+an(2n-1)}{(2a+2n+1)(a+n)}\right) \\
P_4^{(d)} &:= \sum_{i=0}^{d-1} \binom{2n}{i} \frac{B(a+i, a+2n-i)}{B(a,a)} i.
\end{aligned} \tag{42}$$

The sums of the variables defined in Equation 42 in S1 Text for $d = 2n+1$ are,

$$S = a+1, \qquad S_1 = \frac{a+n}{(1+2a)n}, \qquad S_2 = \frac{1}{2a+1}, \qquad S_3 = 0, \qquad S_4 = n. \tag{43}$$

respectively, and with S_3 provided for completeness. The pervasive beta functions in Equation 42 in S1 Text are a consequence of the sampling polynomial implicit in the threshold model (see Equation 3 in the main text and Equation 17 in S1 Text). For example, $P^{(d)}$ is the *cdf* of a beta-binomial random variable parameterized by the number of chromosomes in the GWA study sample $2n$ and the mutation rate a. As we state in the main text, $\mathbb{P}\{\hat{\beta} = \beta\} = 1 - 2P^{(d)}$ when the detection thresholds are both equal to d. The second variable, $P_1^{(d)}$ arises from moments of the form $\mathbb{E}[\bar{X}\hat{\beta}^2]$, the expectation of the product of the mean genotype in the GWA study sample and the effect estimate. The factor $(i-n)/n$ relates the mean genotype $\bar{X}$ to the allele count D, i.e., $\bar{X} = (D-n)/n$. The remaining terms are less immediately interpretable; their rational is implicit in the derivations presented below and the moments provided in Section 13 in S1 Text.

7.1 A form of the polygenic score bias for arbitrary thresholds

The bias of a polygenic score for an individual sampled at time τ in the past and a GWA study conducted at present is,

$$\begin{aligned}
bias(\tau) = \mathbb{E}\left[\hat{Y}(\tau) - Y(\tau)\right] &= \mathbb{E}\left[\hat{C}\right] - C + \sum_{\ell=1}^{L} \mathbb{E}\left[X_\ell(\tau)(\hat{\beta}_\ell - \beta_\ell)\right] + \mathbb{E}\left[\epsilon(\tau)\right] \\
&= \sum_{\ell=1}^{L} \beta_\ell \mathbb{E}\left[\bar{X}_\ell\right] - \mathbb{E}\left[\bar{X}_\ell \hat{\beta}_\ell\right] + \mathbb{E}\left[X_\ell(\tau)\hat{\beta}_\ell\right] - \beta_\ell \mathbb{E}\left[X_\ell(\tau)\right].
\end{aligned} \tag{44}$$

Further simplification of Equation 44 in S1 Text yields Equation 11 in the main text. Using the moments derived in Section 13 in S1 Text, we can simplify Equation 11 in the main text,

$$\begin{aligned} bias_\ell(\tau) &= \beta_\ell \sum_{i=d_{\ell 1}}^{2n-d_{\ell 2}} \binom{2n}{i} \frac{B(a+i, a+2n-i)}{B(a,a)} \left(e^{-a\tau}\left(\frac{i-n}{a+n}\right) - \frac{i-n}{n}\right) \\ &= \beta_\ell \left(e^{-a\tau}\left(\frac{1}{a+n}\right) - \frac{1}{n}\right)\left[n\left(P^{(d_{\ell 1})} - P^{(d_{\ell 2})}\right) - \left(P_4^{(d_{\ell 1})} - P_4^{(d_{\ell 2})}\right)\right] \\ &\approx \beta_\ell \left(e^{-a\tau} - 1\right)\left[\left(P^{(d_{\ell 1})} - P^{(d_{\ell 2})}\right) - \frac{1}{n}\left(P_4^{(d_{\ell 1})} - P_4^{(d_{\ell 2})}\right)\right], \end{aligned} \tag{45}$$

where the last line follows for $a \ll n$. As stated in *Bias* of **Analytical Results** in the main text, for equal mutation rates and symmetric detection thresholds, $bias(\tau)$ is 0 for all τ.

In Fig S1A in S1 Text, we plot $bias_\ell(\tau)$ in the presence of detection asymmetry for a larger range of mutation rates than presented in the main text, $a \in \{10^{-4}, 10^{-3}, 10^{-2}, 1\}$. In addition, we vary the GWA study sample size over three orders of magnitude, $n = \{10^4, 10^5, 10^6\}$. While the mutation rate $a = 1$ is not biologically plausible—this extreme illustrates features of our model that further illuminate, by contrast, the small mutation rate regime. For example, when $a = 1$ the probability of detecting a locus as significant depends heavily on the GWA study sample size n (Fig S1C in S1 Text). Specifically, for $a = 1$, $P^{(d_\ell)} = \frac{d_\ell}{2n}$ increases linearly with d_ℓ. In contrast, for $a \ll 1$ and modest n, this probability is insensitive to n (Fig S1B in S1 Text). Specifically, $P^{(d_\ell)} \approx 0.5$ for all values of n and d_ℓ as most of the allele frequencies are very close to, or equal to zero or one, and thus will always elude detection in GWA studies with finite sample sizes. In other words, once n is large enough, varying d_ℓ yields diminishing returns.

In Fig S1A in S1 Text, we set $d_{\ell 1} = 1$ and $d_{\ell 2} = n$ for each sample size to illustrate the effects of an extreme imbalance. For $d_{\ell 2} = n$, positive effect alleles cannot be detected, while $d_{\ell 1} = 1$ implies that a negative effect allele will be detected as long as it is segregating in the GWA study sample. As $d_{\ell 1} < d_{\ell 2}$, sites where the trait-decreasing allele is at a higher frequency in the GWA study ($D_\ell < n$) will be detected more often than sites where the trait-increasing allele is at a higher frequency ($D_\ell > n$). This implies that sites where the majority of individuals in the GWA study possess negative effect alleles are more likely to have non-zero effects in the genetic prediction. At the same time, the majority of sites contributing to the estimated intercept $\hat{C}$ will have $\bar{X}_\ell > 0$, and thus, in expectation, $\hat{C} \geq 0$. Thus, at $\tau = 0$, the excess positive contributions to the estimated intercept are tempered by the excess negative contributions to the genetic prediction. As τ increases, $bias_\ell(\tau)$ becomes more positive (Fig S1A in S1 Text). This is because the estimated intercept $\hat{C}$ is constant, whereas, the expected value of the genetic prediction approaches zero with increasing τ. The latter follows from the fact that as τ increases, the genotype of the ancient sample $X_\ell(\tau)$ becomes independent of the average genotype in the GWA study $\bar{X}_\ell$, and its expected value approaches zero. Thus, in the large τ limit, $bias(\tau) = \mathbb{E}[\hat{C}]$, which is positive for $d_1 < d_2$.

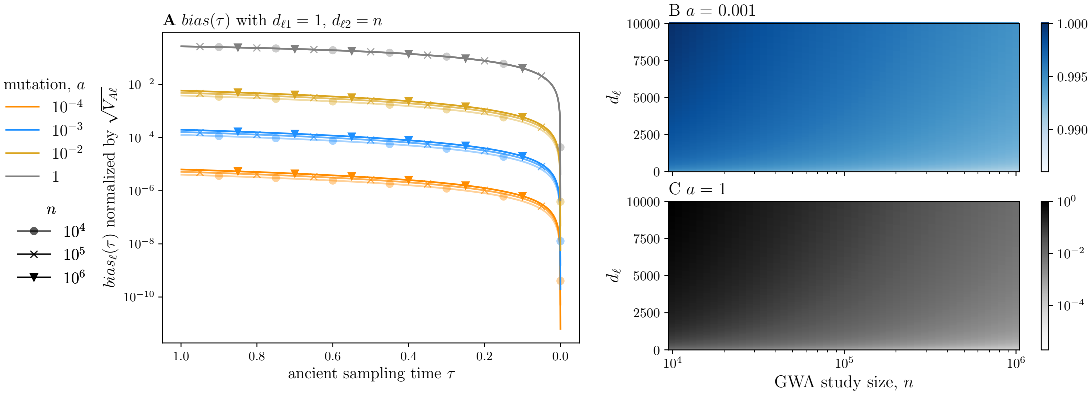

Fig S1. Asymmetry in the detection threshold. In (A), we plot $bias_\ell(\tau)$ for several mutation rates, $a \in \{10^{-4}, 10^{-3}, 10^{-2}, 1\}$ (color) and across three GWA study sizes, $n \in \{10^3, 10^4, 10^5\}$ (shape). We set the detection thresholds to $d_1 = 1$ and $d_2 = n$. In (B) and (C), we plot $2P^{(d)}$ as a function of n and the detection threshold d for $a = 10^{-3}$ (blue) and $a = 1$ (gray), respectively.

Approximating the increase of $bias_\ell(\tau)$, given in Equation 45 in S1 Text, for small a and large n,

$$bias_\ell(\tau) \approx bias_\ell(0) + \beta_\ell a \tau \left(P^{(d_{\ell 1})} - P^{(d_{\ell 2})}\right), \tag{46}$$

gives us additional insight into these results. In Equation 46 in S1 Text, $bias_\ell(0)$ is an exact expression for the $bias_\ell(\tau)$ evaluated at $\tau = 0$; and $P^{(d_{\ell i})}$ is the probability that the allele count in the GWA study, D_ℓ, is less than $d_{\ell i}$ for $i = 1, 2$ (Equation 42 in S1 Text). Under our assumptions, $P^{(d_{\ell i})}$ is the cumulative distribution function (*cdf*) of a beta-binomial random variable with $2n$ trials, parameterized by the mutation rate a. At $\tau = 0$, the focal individual is an independent sample from the GWA study population. Thus, the intercept term captures contributes to $bias_\ell(\tau)$ exclusively due to finite sampling. For $\tau > 0$, allelic turnover induces changes in the frequencies of sites not detected in the GWA study (ℓ such that $\hat{\beta}_\ell = 0$), which may contribute to the phenotypic variation of ancient individuals. Thus, the linear term captures additional bias due to finite sampling *and* allelic turnover.

We conclude that increases in $bias_\ell(\tau)$ with τ depend primarily on the difference in the detection probabilities for trait-increasing and decreasing alleles, i.e., $P^{(d_{\ell 1})} - P^{(d_{\ell 2})}$. For small a, this difference is small relative to the (square root of the) additive genetic variance V_A due to the fact that the detection probability is insensitive to the threshold d_ℓ. However, differences in sample size are apparent when the mutation rate is small—with larger sample sizes yielding a larger bias (Fig S1A in S1 Text). This sample size dependency is due to the fact that increased power to detect low frequency alleles with larger n results in a larger difference between the one-sided detection probabilities. As a approaches one, the effects of sample size diminish (in log scale). For $a = 1$, the difference in one-sided detection probabilities is $P^{(1)} - P^{(n)} = \frac{n-1}{2n}$, which will be close to $\frac{1}{2}$ for modest values of n. In addition, for large a, $bias_\ell(\tau)$ is non-negligible relative to (the square root of) $\mathbb{E}[V_A]$.

7.2 Mean-squared error

Substituting the definitions of $\hat{Y}(\tau)$ and $Y(\tau)$, we can simplify the expression for the mean-squared error (mse),

$$\begin{aligned} mse(\tau) = \mathbb{E}\left[\left(\hat{Y}(\tau) - Y(\tau)\right)^2\right] &= \mathbb{E}\left[\left((\hat{C} - C) + \sum_{\ell=1}^{L} X_\ell(\hat{\beta}_\ell - \beta_\ell) - \epsilon(\tau)\right)^2\right] \\ &= \mathbb{E}\left[\left(\sum_{\ell=1}^{L}(X_\ell(\tau) - \bar{X}_\ell)(\hat{\beta}_\ell - \beta_\ell) + (\bar{\epsilon} - \epsilon(\tau))\right)^2\right] \\ &= \sum_{\ell=1}^{L} \mathbb{E}\left[(X_\ell(\tau) - \bar{X}_\ell)^2(\hat{\beta}_\ell - \beta_\ell)^2\right] + \mathbb{E}\left[(\bar{\epsilon} - \epsilon(\tau))^2\right], \end{aligned} \tag{47}$$

where the cross-terms in Equation 47 in S1 Text cancel due to independence between the environmental noise, which has mean 0, and the genotypes. The error term simplifies,

$$\mathbb{E}\left[(\bar{\epsilon} - \epsilon(\tau))^2\right] = \mathbb{E}\left[\bar{\epsilon}^2 - 2\bar{\epsilon}\epsilon(\tau) + (\epsilon(\tau))^2\right] = \left(\frac{n-1}{n}\right)\sigma_e^2. \tag{48}$$

When $\sigma_e^2 = 0$, the mse reduces to,

$$\begin{aligned} mse_\ell(\tau) = &\ \mathbb{E}\left[X_\ell^2(\tau)\hat{\beta}_\ell^2\right] - 2\beta_\ell\mathbb{E}\left[X_\ell^2(\tau)\hat{\beta}_\ell\right] + \beta_\ell^2\mathbb{E}\left[X_\ell^2(\tau)\right] \\ &- 2\left(\mathbb{E}\left[X_\ell(\tau)\bar{X}_\ell\hat{\beta}_\ell^2\right] - 2\beta_\ell\mathbb{E}\left[X_\ell(\tau)\bar{X}_\ell\hat{\beta}_\ell\right] + \beta_\ell^2\mathbb{E}\left[X_\ell(\tau)\bar{X}_\ell\right]\right) \\ &+ \mathbb{E}\left[\bar{X}_\ell^2\hat{\beta}_\ell^2\right] - 2\beta_\ell\mathbb{E}\left[\bar{X}_\ell^2\hat{\beta}_\ell\right] + \beta_\ell^2\mathbb{E}\left[\bar{X}_\ell^2\right]. \end{aligned} \tag{49}$$

For equal detection thresholds $d_{\ell 1} = d_{\ell 2} = d_\ell$, and with substitution of the variables defined in Equation 42 in S1 Text, Equation 49 in S1 Text reduces to,

$$mse(\tau) = 2\sum_{\ell=1}^{L}\beta_\ell^2\left[\left(\frac{a+1}{2a+1}\right)P^{(d_\ell)} + P_1^{(d_\ell)} - 2e^{-a\tau}P_2^{(d_\ell)} + \left(\frac{1}{2a+1}\right)e^{-(2a+1)\tau}P_3^{(d_\ell)}\right], \tag{50}$$

where $d_\ell = \lceil n\gamma_\ell \rceil$. The change in $mse(\tau)$ is due to the difference between the two exponential terms in Equation 50 in S1 Text. From Equation 50 in S1 Text, we derive the derivative of $mse(\tau)$,

$$\frac{dmse(\tau)}{d\tau} = 2\sum_{\ell=1}^{L}\beta_\ell^2\left[2aP_2^{(d_\ell)}e^{-a\tau} - P_3^{(d_\ell)}e^{-(2a+1)\tau}\right], \tag{51}$$

which, for small a and τ, is,

$$\frac{dmse(\tau)}{d\tau} \approx 2\sum_{\ell=1}^{L}\beta_\ell^2\left[2aP_2^{(d_\ell)} - P_3^{(d_\ell)}e^{-\tau}\right] \approx 2a\sum_{\ell=1}^{L}\beta_\ell^2 P^{(d_\ell)}\left(2 - e^{-\tau}\right). \tag{52}$$

7.3 Expected additive genetic variance

We solve for $\hat{V}_A(\tau)$ in an ancient sample of size n_a and a GWA study sample of size n. Considering a single locus ℓ and conditioning on the ancient and contemporary allele frequencies,

$$\begin{aligned}\hat{V}_{A\ell}(\tau) &= 2\mathbb{E}\left[\mathbb{E}\left[\hat{\beta}_\ell^2 \hat{Z}_\ell(\tau)(1-\hat{Z}_\ell(\tau))|Z_\ell, Z_\ell(\tau)\right]\right] \\ &= 2\mathbb{E}\left[\mathbb{E}\left[\hat{Z}_\ell(\tau)(1-\hat{Z}_\ell(\tau))|Z_\ell(\tau)\right]\mathbb{E}\left[\hat{\beta}_\ell^2|Z_\ell\right]\right] \\ &= 2\left(\frac{2n_a-1}{2n_a}\right)\beta_\ell^2 \mathbb{E}\left[Z_\ell(\tau)(1-Z_\ell(\tau))\wp(Z_\ell, d_\ell, n)\right],\end{aligned} \tag{53}$$

where $\wp(Z_\ell, 2n, d_\ell)$ is the Binomial sampling probability defined in Equation 18 in S1 Text. Substituting the spectral representation of the *tdf* yields,

$$\hat{V}_{A\ell}(\tau) = \beta_\ell^2\left(\frac{2n_a-1}{2n_a}\right)\left(\frac{1}{2a+1}\right)\left[a(1-2P^{(d_\ell)}) + 2e^{-(2a+1)\tau}P_3^{(d_\ell)}\right]. \tag{54}$$

To compare $\hat{V}_A$ across parameter regimes, we normalize by the expected population additive genetic variance $\mathbb{E}[V_A]$. At stationarity and for mutation rate a,

$$\mathbb{E}\left[V_{A\ell}(\tau)\right] = 2\beta_\ell^2 \int_{z_\tau} z_\tau(1-z_\tau)\kappa(z_\tau)dz_\tau = \left(\frac{a}{2a+1}\right)\beta_\ell^2, \tag{55}$$

where $\kappa(\cdot)$ is the stationary density given in Equation 1 in S1 Text.

For small a, $\hat{V}_A(\tau)$ will change at rate,

$$\frac{d\mathbb{E}[\hat{V}_A(\tau)]}{d\tau} \approx -2\left(\frac{2n_a-1}{2n_a}\right)e^{-\tau}\sum_{\ell=1}^{L}\beta_\ell^2 P_3^{(d_\ell)} \approx -2\left(\frac{2n_a-1}{2n_a}\right)e^{-\tau}a\sum_{i=1}^{L}\beta_\ell^2 P^{(d_\ell)}, \tag{56}$$

where the right hand expression follows from the approximation $P_3^{(d_\ell)} \approx aP^{(d_\ell)}$ (Section 9 in S1 Text).

7.4 Approximate sample correlation coefficient

In this subsection, we (i) describe the two approximation steps implicit in our definition of $\rho^2(\tau)$ given in Equation 10 in the main text; (ii) derive an explicit form for $\rho^2(\tau)$; and (iii) derive the approximate decay of relative accuracy $\rho^2(\tau)/\rho^2(0)$.

(i) A practitioner is often interested in the accuracy of their predictor with respect to a particular sample. This sample correlation coefficient (for n_a ancient individuals from τ) is defined as,

$$r(\tau) := \frac{Cov[\hat{\boldsymbol{Y}}(\tau), \boldsymbol{Y}(\tau)]}{\sqrt{Var[\hat{\boldsymbol{Y}}]Var[\boldsymbol{Y}]}} = \frac{\sum_{i=1}^{n_a}(\hat{Y}_i(\tau)-\bar{\hat{Y}}(\tau))(Y_i(\tau)-\bar{Y}(\tau))}{\sqrt{\sum_{i=1}^{n_a}(\hat{Y}_i(\tau)-\bar{\hat{Y}}(\tau))^2}\sqrt{\sum_{i=1}^{n_a}(Y_i(\tau)-\bar{Y}(\tau))^2}}, \tag{57}$$

where $Cov[\cdot,\cdot]$ and $Var[\cdot]$ are the sample covariance and variance operators, respectively; and $\hat{\boldsymbol{Y}}(\tau), \boldsymbol{Y}(\tau) \in \mathbb{R}^{n_a}$ are the n_a-dimensional vectors of polygenic scores and phenotypes with sample means $\bar{\hat{Y}}(\tau)$ and $\bar{Y}(\tau)$, respectively. Ultimately, we will approximate the expectation of the squared sample correlation coefficient $r^2(\tau)$ with a ratio of expectations,

$$\mathbb{E}\left[r^2(\tau)\right] \approx \frac{\mathbb{E}\left[Cov[\hat{\boldsymbol{Y}}(\tau), \boldsymbol{Y}(\tau)]\right]^2}{\mathbb{E}[Var[\hat{\boldsymbol{Y}}(\tau)]]\mathbb{E}\left[Var[\boldsymbol{Y}(\tau)]\right]}, \tag{58}$$

which we defined as $\rho^2(\tau)$ in Equation 10 in the main text. To arrive at this approximation, we must first approximate the expectation of the ratio in Equation 57 in S1 Text as the ratio of expectations. Second, we must pull the expectation inside the square roots in the denominator. A full investigation of the validity of these steps in general is beyond the scope of the present study. Rather, we validate these approximation steps by simulations of a few parameter regimes of particular interest.

After these two approximation steps, we compute the quantity in Equation 58 in S1 Text exactly under our framework. We take each element of Equation 58 in S1 Text in turn.

(ii) When we plug in our modeling assumptions, the numerator of Equation 57 in S1 Text becomes

$$Cov[\hat{\boldsymbol{Y}}(\tau), \boldsymbol{Y}(\tau)] = \sum_{i=1}^{n_a} \sum_{\ell,\ell'} \hat{\beta}_\ell \beta_{\ell'} (X_{i\ell}(\tau) - \bar{X}_\ell(\tau))(X_{i\ell'}(\tau) - \bar{X}_{\ell'}(\tau)) + Cov[\hat{\boldsymbol{Y}}(\tau), \boldsymbol{\epsilon}], \tag{59}$$

due to the linearity of the covariance operator, with $\boldsymbol{\epsilon} \in \mathbb{R}_a^n$ as the vector of environmental effects. In expectation, assuming *iid* loci with equal effects β and *iid* ancient samples,

$$\mathbb{E}\left[Cov[\hat{\boldsymbol{Y}}(\tau), \boldsymbol{Y}(\tau)]\right] = \frac{1}{n_a} \sum_{i=1}^{n_a} \sum_{\ell=1}^{L} \beta_\ell \mathbb{E}\left[\hat{\beta}_\ell (X_{i\ell}(\tau) - \bar{X}_\ell(\tau))^2\right] = L\beta \mathbb{E}\left[\hat{\beta}(X_i(\tau) - \bar{X}(\tau))^2\right]. \tag{60}$$

Similarly,

$$\mathbb{E}\left[Var[\hat{\boldsymbol{Y}}(\tau)]\right] = L\mathbb{E}\left[\hat{\beta}^2 (X_i(\tau) - \bar{X}(\tau))^2\right], \tag{61}$$

which, under our simple threshold model is equal to the expectation of the covariance given in Equation 60 in S1 Text. Finally,

$$\mathbb{E}\left[Var[\boldsymbol{Y}(\tau)]\right] = L\beta^2 \mathbb{E}\left[(X(\tau) - \bar{X}(\tau))^2\right] + \left(\frac{n_a - 1}{n_a}\right) \sigma_e^2. \tag{62}$$

All together, our approximation for $r^2(\tau)$ reduces to,

$$\mathbb{E}\left[r^2\right] \approx \frac{L\beta \mathbb{E}\left[\hat{\beta}(X(\tau) - \bar{X}(\tau))^2\right]}{L\beta^2 \mathbb{E}\left[(X(\tau) - \bar{X}(\tau))^2\right] + \left(\frac{n_a-1}{n_a}\right)\sigma_e^2} = \frac{\mathbb{E}\left[\hat{\beta}(X(\tau) - \bar{X}(\tau))^2\right]/\beta}{\mathbb{E}\left[(X(\tau) - \bar{X}(\tau))^2\right] + \left(\frac{n_a-1}{n_a}\right)\sigma_{e'}^2}, \tag{63}$$

where $\sigma_{e'}^2 = \sigma_e^2/(L\beta^2)$. All that remains is to solve for the expectations in the numerator and denominator of Equation 63 in S1 Text. The first involves both the GWA study and ancient sample times,

$$\begin{aligned}
\mathbb{E}\left[\hat{\beta}(X(\tau) - \bar{X}(\tau))^2\right] &= \mathbb{E}\left[\mathbb{E}\left[\hat{\beta}(X(\tau) - \bar{X}(\tau))^2 | Z(0), Z(\tau)\right]\right] \\
&= \beta \mathbb{E}\left[\wp(Z(0)) \mathbb{E}\left[(X(\tau) - \bar{X}(\tau))^2 | Z(\tau)\right]\right] \\
&= 2\beta \left(\frac{n_a - 1}{n_a}\right) \mathbb{E}\left[\wp(Z(0)) Z(\tau)(1 - Z(\tau))\right],
\end{aligned} \tag{64}$$

which we recognize as closely related to the expected estimated additive genetic variance, $\hat{V}_A(\tau)$, with $\wp(Z(0))$ defined in Equation 18 in S1 Text. The expectation in the denominator is,

$$\mathbb{E}\left[\hat{\beta}(X(\tau) - \bar{X}(\tau))^2\right] = 2\left(\frac{n_a - 1}{n_a}\right) \mathbb{E}\left[Z(\tau)(1 - Z(\tau))\right] = \left(\frac{n_a - 1}{n_a}\right)\left(\frac{a}{2a + 1}\right), \tag{65}$$

which is equal to the $\mathbb{E}[V_A]$ at stationarity normalized by the squared true effect β^2 and multiplied by the n_a-dependent factor to account for ancient sample size. We then see that our approximation to the expectation of $r^2(\tau)$ is insensitive to the ancient sample size and equal to,

$$\mathbb{E}\left[r^2(\tau)\right] \approx \rho^2(\tau) := \frac{2\mathbb{E}\left[\wp(Z(0))Z(\tau)(1-Z(\tau))\right]}{\frac{a}{2a+1}+\sigma_{e'}^2} = \left(\frac{2n_a}{2n_a-1}\right)\frac{\hat{V}_{A\ell}(\tau)/\beta^2}{\frac{a}{2a+1}+\sigma_{e'}^2}, \tag{66}$$

where the ancient sample size dependent factor in the rightmost expression cancels with its inverse in $\hat{V}_{A\ell}(\tau)$. Thus, the sample correlation coefficient is proportional to the estimated additive genetic variance, and its derivative is given by,

$$\begin{aligned}\frac{d\mathbb{E}\left[r^2(\tau)\right]}{d\tau} &\approx \frac{d}{d\tau}\left(\frac{2n_a}{2n_a-1}\right)\frac{\hat{V}_{A\ell}(\tau)/\beta^2}{\frac{a}{2a+1}+\sigma_{e'}^2} \\ &\approx \left(\frac{1}{\frac{a}{2a+1}+\sigma_{e'}^2}\right)\left(\frac{2}{2a+1}\right)(-(2a+1))e^{-(2a+1)\tau}P_3^{(d)} \\ &\approx \left(\frac{1}{a+\sigma_{e'}^2}\right)2e^{-\tau}P_3^{(d)} \approx \left(\frac{a}{a+\sigma_{e'}^2}\right)2e^{-\tau}P^{(d)},\end{aligned} \tag{67}$$

where the last line follows for $a \ll 1$, as $e^{-(2a+1)\tau} \approx e^{-\tau}$ and $P_3^{(d)} \approx aP^{(d)}$ (Equation 81 in S1 Text).

(iii) We show that for small mutation rates, relative accuracy decays at a rate that is independent of the mutation rate a and detection threshold d. For *iid* loci,

$$\rho^2(\tau)/\rho^2(0) = \frac{a(1-2P^{(d)})+2e^{-(2a+1)\tau}P_3^{(d)}}{a(1-2P^{(d)})+2P_3^{(d)}} \approx \frac{2e^{-\tau}P_3^{(d)}}{2P_3^{(d)}} = e^{-\tau}, \tag{68}$$

where, we have claimed that $a(1-2P^{(d)}) \approx 0$ for all $d \in \{1,\ldots,n\}$, and that $2a+1 \approx 1$. If we relax the *iid* assumption, we have,

$$\rho^2(\tau)/\rho^2(0) = \frac{\sum_{\ell=1}^{L}\beta_\ell^2\left[a(1-2P^{(d_\ell)})+2e^{-(2a+1)\tau}P_3^{(d_\ell)}\right]}{\sum_{\ell=1}^{L}\beta_\ell^2\left[a(1-2P^{(d_\ell)})+2P_3^{(d_\ell)}\right]}, \tag{69}$$

which could be computed for a given distribution of β_ℓ. For $a \ll 1$, the $a(1-2P^{(d_\ell)})$ terms in Equation 69 in S1 Text *may be* negligible, yielding the same result as Equation 68 in S1 Text, which implies that relative accuracy is insensitive to distributional assumptions on β for small a. However, more rigorous theoretical and simulation-based work is required to assess the accuracy of this claim.

8 Expected accuracy in the UK Biobank

Our theory characterizes ancient polygenic scores in a highly idealized setting. Namely, the population size is constant, allele frequencies evolve neutrally at stationarity, and the estimation of effects coheres with a simple threshold model. In addition, we provide statistics parameterized by a single fixed effect size (although see Section 7.4 in S1 Text where we begin to relax this assumption). In practice, many of these assumptions are likely violated. For example, human populations have undergone numerous population size changes, including both bottlenecks and expansions, as

well as admixture events [10]. In addition, many human traits are thought to be under some form of selection, which necessarily alters the allele frequency dynamics of causal loci and neutral loci nearby (e.g., [11–13]). And, confounding factors like population structure may still complicate interpretation of the results of GWA studies [14,15]. Lastly, causal effect sizes of complex traits, e.g. height, vary across loci. This distribution of effects is difficult to characterize, likely with significant mass near zero (e.g., see [16]).

In this section, we tackle the last of these complications: that effect sizes are different at each locus, while still retaining the other simplifying assumptions. We model the variation among effect sizes by assuming that each effect is random and *iid*, i.e., independent and drawn from the same probability distribution. We do not attempt to estimate this distribution from the UK Biobank summary statistics, as for example in [16–18]. Instead, we consider several parameterizations of the causal effect size distribution, all with ample mass near zero, including a distribution previously estimated from GWA study summary statistics in [18].

Using the UK Biobank summary statistics, we estimate the relationship between the minimum allele frequency required to detect a single nucleotide polymorphism (SNP) with an effect size β as significant under a particular significance threshold α. In essence, we are replacing our theoretical parameterization of the per-locus detection threshold d_ℓ (Equation 11 in S1 Text) with one derived from data. Then, assuming the population is at equilibrium, we compute the approximate decay in accuracy, as measured by $\rho^2(\tau)$ in Equation 10 in the main text, for each of the causal distributions and arbitrary ancient sampling times.

Causal effect size distributions. As the causal effect size distribution for human height is unknown, we consider several potential distributions (Fig S2 in S1 Text). Namely, we model the absolute values of the effect sizes $|\beta|$ as (i) exponential random variables with rate $\lambda \in \{10, 100, 500\}$, referred to as $f_{\text{exp}}(\cdot;\lambda)$; (ii) Gamma distributed random variables with shape parameter $\lambda \in \{10^{-3}, 0.5\}$ and scale parameter equal to one, referred to as $f_\gamma(\cdot;\lambda)$; and (iii) a mixture of folded normal distributions estimated from GWA study summary statistics in [18],

$$f_{\text{mix}}(b) = 0.9 \cdot \mathcal{N}^*(b; 0, 1.5439 \cdot 10^{-5}) + 0.1 \cdot \mathcal{N}^*(b; 0, 2.021 \cdot 10^{-4}), \tag{70}$$

for some effect size b, where $\mathcal{N}^*(b; 0, \sigma^2)$ denotes the likelihood of effect size b under a folded normal distribution with mean zero and variance σ^2. In all cases, we discretize these distributions over a set $\boldsymbol{\beta}$ of 5000 linearly spaced values in the range $[10^{-4}, 0.1]$. In doing so, we are excluding very large effect mutations—such mutations would be more likely to substantially deviate from neutral dynamics—and mutations with effects indistinguishable from zero.

Estimating the relationship between effect size and the detection threshold. We use the height summary statistics to estimate a function relating effect size to the allele frequency detection threshold, denoted by $g_\alpha(\cdot)$. The function $g_\alpha(b)$ specifies the minimum allele frequency required to detect an effect of size b as non-zero under a given significance threshold α. Here, we use $\alpha = 10^{-8}$ to account for the multiple testing burden imposed by conducting approximately 12 million association tests. We compute the minimum effect size detected as significant (p-value $\leq \alpha$) among SNPs within 250 non-overlapping, log-spaced allele frequency bins, and subsequently interpolate between these minima to specify $g_\alpha(\cdot)$ over the continuous interval $[10^{-3}, 0.5]$. Finally, we "smooth" this function by forcing it to be non-increasing.

Computing the accuracy metrics. An effect size distribution (described above) coupled with our equilibrium assumption specifies the expected additive genetic variance, V_A. For a population-

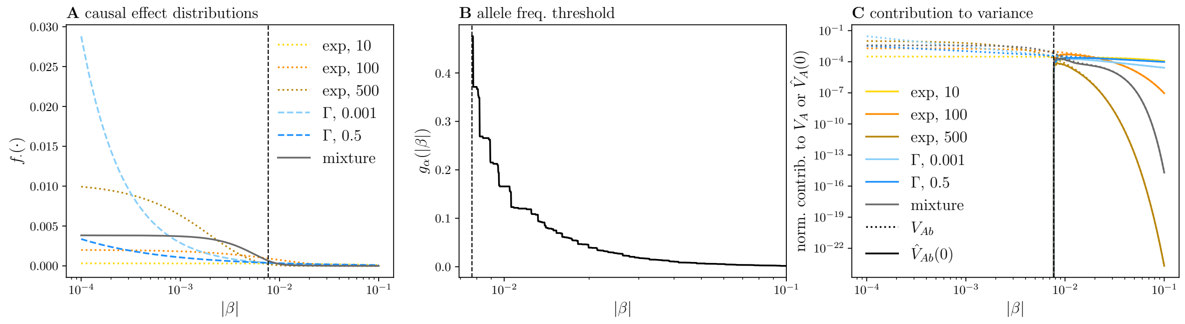

Fig S2. Effect size distribution. In (A), we plot the six causal distributions described above. The dashed vertical line here, and in (B) and (C), indicates the minimum effect size that can be detected, as specified by $g_\alpha(\cdot)$ of (B). In (B), we find the minimum allele frequency $g_\alpha(\beta)$ required to detect an effect of size β using the significance threshold $\alpha = 10^{-8}$ and a set of 273,671 SNPs with p-values $\leq \alpha$. And, in (C), we plot the relationship between a SNP's effect size and its contribution to the expected additive genetic variance V_{Ab} (dotted lines) or the estimated additive genetic variance at the time of the GWA study $\hat{V}_{Ab}(0)$ (solid lines) for each of the causal distributions. Both values are normalized by the squared effect size β^2 and the expected genetic variance at stationarity, $a/(2a+1)$.

scaled mutation rate of a,

$$\mathbb{E}[V_A] = \sum_{b \in \boldsymbol{\beta}} \left(\frac{a}{2a+1} \right) b^2 f.(b), \tag{71}$$

where the sum is over all discretized effect sizes b in the set of effect sizes $\boldsymbol{\beta}$; $a/(2a+1)$ is the expected genetic variance at stationarity (Section 13 in S1 Text); and $f.(\cdot)$ is one of the effect size distributions described above. We can then compute the approximate sample correlation coefficient $\rho^2(\tau)$. In particular, following Equation 19 in the main text and ignoring the ancient sample size dependent factor in the GWA study,

$$\rho^2(0) = \frac{\hat{V}_A(0)}{V_A + \sigma_e^2} = \frac{\sum_{b \in \beta} \hat{V}_{Ab}(0) f.(b)}{\sum_{b \in \beta} \left(\frac{a}{2a+1} \right) b^2 f.(b) + \sigma_e^2}, \tag{72}$$

and following Equation 54 in S1 Text,

$$\hat{V}_{Ab}(0) = \mathbb{E}\left[2\hat{\beta}^2 Z(0)(1 - Z(0)) | \beta = b\right] = b^2 \left(\frac{1}{2a+1} \right) \left[a(1 - 2P^{(d_b)}) + 2P_3^{(d_b)} \right], \tag{73}$$

where $d_b = \lceil 2n g_\alpha(b) \rceil$ is the allele count threshold derived from the function $g_\alpha(\cdot)$, and with $P^{(\cdot)}$ and $P_3^{(\cdot)}$ defined in Equation 42 in S1 Text. Importantly, the denominator of Equation 72 in S1 Text includes non-zero contributions from SNPs that may not achieve genome-wide significance (unequivocally those SNPs with effect sizes to the left of the dashed lines in Fig S2 in S1 Text).

When narrow-sense heritability $h^2 = 0.5$, the environmental variance σ_e^2 is equal to V_A. For an arbitrary ancient sampling time τ,

$$\rho^2(\tau) = \frac{\sum_{b \in \beta} \hat{V}_{Ab}(\tau) f.(b)}{2 \sum_{b \in \beta} \left(\frac{a}{2a+1} \right) b^2 f.(b)} = \frac{\sum_{b \in \beta} \left[a(1 - 2P^{(d_b)}) + 2e^{-(2a+1)\tau} P_3^{(d_b)} \right] b^2 f.(b)}{2a \sum_{b \in \beta} b^2 f.(b)}, \tag{74}$$

where d_b, defined in Equation 73 in S1 Text, is the empirically estimated allele count threshold corresponding to an effect of size b. We compute Equation 74 in S1 Text for each of the six effect size

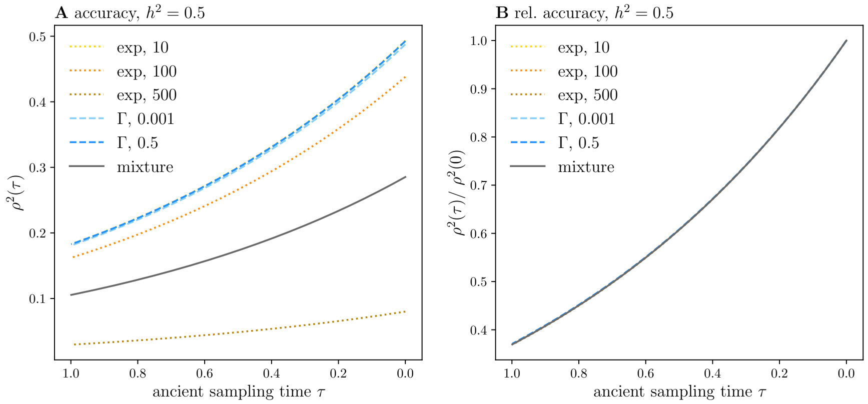

Fig S3. Accuracy and relative accuracy. In (A), we plot the approximate sample correlation coefficient, or polygenic score accuracy $\rho^2(\tau)$, as a function of ancient sampling time τ. We assume the effect size distributions described above and associated allele frequency thresholds (Fig S2 in S1 Text), as well as narrow-sense heritability $h^2 = 0.5$. Relative accuracy is similarly plotted in (B). Note the difference in y-axis limits between panels.

distributions. While not shown, we can similarly compute the other accuracy metrics (normalized by V_A as we do not know the number of causal sites L).

Not unexpectedly, Fig S3 in S1 Text shows that the shape of the causal distribution influences accuracy. However, relative accuracy is indistinguishable across the different distributions. Thus, as observed in *Polygenic score accuracy* in **Analytical Results** of the main text (and speculated on in Section 7.4 in S1 Text), relative accuracy appears insensitive to assumptions on the effect size distribution.

It is important to note that several of these distributions yield predicted accuracies for contemporary samples that overestimate the observed prediction accuracy for height in the UK Biobank sample, for example, estimated to be 0.193 in [7]. This discrepancy suggests that these distributions may not be good approximations to reality. Though, we note that $f_{\text{mix}}(\cdot)$, the distribution estimated in [18], yields the best approximation to observed prediction accuracy in the present day sample.

Nonetheless, many of our simplifying assumptions caution against overinterpretation of these results. In particular, our assumption of neutrality implies that alleles are *iid* irrespective of the magnitudes of their effects; loci may also not be at stationarity. Indeed, large effect alleles are likely to be more deleterious and thus subject to stronger selection relative to small effect alleles [11, 19, 20]. Nonetheless, the application of our theory in this context provides insight into the relationship between the causal effect size distribution and prediction accuracy. Furthermore, we provide some preliminary evidence that relative accuracy may be more robust to violations of at least some of our assumptions.

9 Deriving approximations to the metrics

In the main text, we present several approximations for the initial rate of increase or decrease of the metrics. Here, we show how we arrived at these approximations from the exact forms given in the previous sections. For a given metric, we first compute a first order Taylor series expansion (in τ). We then find the intercept, i.e., the value of the statistic at zero, and the slope. We subsequently make use of the following approximations: (i) $P_1^{(d)} \approx P^{(d)}$; (ii) $P_2^{(d)} \approx P^{(d)}$; and (iii) $P_3^{(d)} \approx aP^{(d)}$.

Approximate metrics. For the *bias*, this approach yields,

$$\begin{aligned} bias_\ell(\tau) &\approx \beta_\ell \left[(1-a\tau)\left(\frac{1}{a+n}\right) - \frac{1}{n}\right]\left[n\left(P^{(d_{\ell 1})} - P^{(d_{\ell 2})}\right) + \left(P_4^{(d_{\ell 2})} - P_4^{(d_{\ell 1})}\right)\right] \\ &\approx bias_\ell(0) + \beta_\ell \cdot a\tau \left(P^{(d_{\ell 1})} - P^{(d_{\ell 2})}\right), \end{aligned} \tag{75}$$

where the last line follows from (i) using the approximations noted in the prelude; (ii) ignoring the $P_4^{(d_{\ell i})}$ terms in the slope (which are order $\mathcal{O}(\frac{1}{n})$); (iii) and $\frac{1}{a+n} \approx \frac{1}{n}$. Using the same approach, $mse_\ell(\tau)$ with equal thresholds d_ℓ, becomes,

$$\begin{aligned} mse_\ell(\tau) &\approx 2\beta_\ell^2 \Big[\left(\frac{a+1}{2a+1}\right) P^{(d_\ell)} + P_1^{(d_\ell)} - 2P_2^{(d_\ell)} + \left(\frac{1}{2a+1}\right) P_3^{(d_\ell)} + 2a\tau P_2^{(d_\ell)} - \tau P_3^{(d_\ell)}\Big] \\ &\approx mse_\ell(0) + 2\beta_\ell^2 a\tau P^{(d_\ell)}. \end{aligned} \tag{76}$$

And, we can approximate $\hat{V}_A(\tau)$ as,

$$\begin{aligned} \hat{V}_{A\ell}(\tau) &\approx \left(\frac{2n_a - 1}{2n_a}\right) \beta_\ell^2 \left(\frac{1}{2a+1}\right) \left[\left(a(1 - 2P^{(d_\ell)}\right) + \left(2\left(1 - (2a+1)\tau\right) P_3^{(d_\ell)}\right)\right] \\ &\approx \hat{V}_{A\ell}(0) - 2\left(\frac{2n_a - 1}{2n_a}\right)\beta_\ell^2 a P^{(d_\ell)}\tau. \end{aligned} \tag{77}$$

The approximation for the accuracy $\rho^2(\tau)$ follows immediately from Equation 77 in S1 Text.

In Figs S4A and S4D in S1 Text, we plot the approximate rate of change $2aP^{(d)}$, for low and high detection thresholds. In addition, in Fig S5 in S1 Text, we compare our exact theoretical results to their approximations over a short time scale of $\tau \in [0.2, 0]$. We observe that the approximation fares better for smaller values of d, as well as, for larger n. Below, we show how some of the steps in our approximations are adversely affected by large d and small n.

Approximation error. We quantify the error incurred in the approximation $P_1^{(d)} \approx P_3^{(d)}$ and $P_2^{(d)} \approx P_3^{(d)}$. To do so, we first express $P_1^{(d)}$ and $P_2^{(d)}$ as functions of $P_3^{(d)}$. We refer to the i-th term in quantities specified in Equation 42 in S1 Text as $P_\cdot^i$, dropping the superscript d for succinctness.

$$P_1^i = \left(\frac{i-n}{n}\right)^2 \binom{2n}{i} \frac{B(a+i, a+2n-i)}{B(a,a)} = \left(\frac{i-n}{n}\right)^2 P^i = \left(\frac{i}{n}\right)^2 P^i - 2\left(\frac{in}{n^2}\right) + P^i. \tag{78}$$

Thus,

$$P_1^{(d)} = P^{(d)} + \frac{1}{n^2}\sum_{i=0}^{d-1} i^2 P^i - \frac{2}{n}\sum_{i=0}^{d-1} iP^i. \tag{79}$$

Note that the summations in Equation 79 in S1 Text are the second and first moments of a beta-binomial random variable truncated at $d-1$, respectively. As long as the two summations are $O(n)$ and $O(1)$, respectively, the error of the approximation will be smaller than $O(\frac{1}{n})$ as both summations are non-negative. We can repeat the same procedure for $P_2^{(d)}$,

$$P_2^{(d)} = \left(\frac{n}{a+n}\right) P^{(d)} + \frac{1}{n(a+n)} \sum_{i=0}^{d-1} i^2 P^i - \left(\frac{2}{a+n}\right) \sum_{i=0}^{d-1} iP^i \approx P_1^{(d)}, \tag{80}$$

where the approximation is valid for $a \ll n$, and in this regime, our analysis in the previous paragraph also applies to $P_2^{(d)}$.

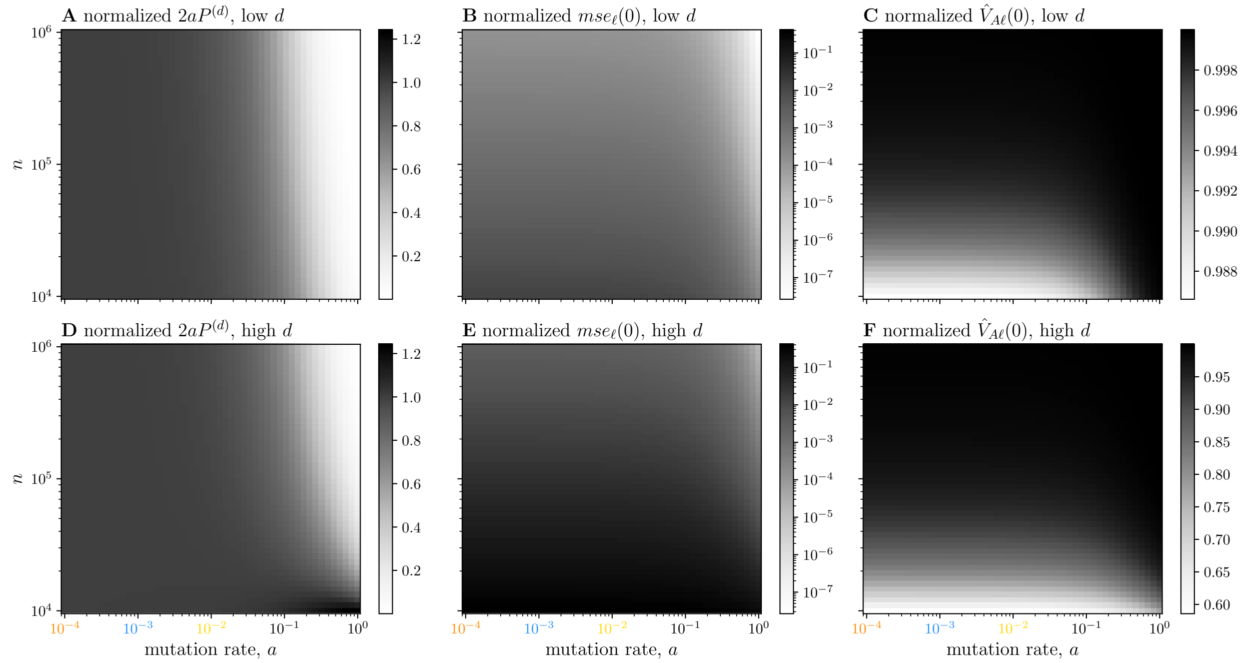

Fig S4. Approximations to the mean-squared error and expected estimated additive genetic variance. In (A), we plot $2aP^{(d)}$ normalized by $\mathbb{E}[V_A] = \beta^2(\frac{a}{2a+1})$ across a range of mutation rates $a \in \{10^{-4}, \dots, 1\}$ and GWA study sample sizes n, for a small detection threshold. Here, d is either 132 or 133, corresponding to a squared effect size of $\beta^2 = 0.25$, when the significance threshold is $\alpha = 10^{-8}$ and the phenotypic variance $V_p = 1$. In (B) and (C), we plot the initial $mse_\ell(\tau)$ and $\hat{V}_{A\ell}(\tau)$ (both normalized by the true V_A) for small d. In (D-F), we repeat plots (A-C), except with a higher detection threshold, respectively. The smaller effect size of $\beta^2 = 0.01$ yields thresholds in the range $d \in \{3209, \dots, 4142\}$, in order of increasing sample size. Note that in contrast to the other pairs of plots, (C) and (F) do not share a scale.

Finally, we consider the approximation $P_3^{(d)} \approx aP^{(d)}$,

$$\begin{aligned}
P_3^i &= P^i\left(\frac{(2a+1)i(i-2n)+an(2n-1)}{(2a+2n+1)(a+n)}\right) + aP^i - aP^i \\
&= aP^i + P^i\left(\frac{(2a+1)i(i-2n)+an(2n-1)-a(2a+2n+1)(a+n)}{(2a+2n+1)(a+n)}\right) \\
&= aP^i + P^i\left(\frac{(2a+1)i(i-2n)-a(2a^2+4an+a+2n)}{(2a+2n+1)(a+n)}\right) \\
&= aP^i + P^i\left(\frac{(2a+1)i(i-2n)-a(2a+1)(a+2n)}{(2a+2n+1)(a+n)}\right) \\
&= aP^i + (2a+1)P^i\left(\frac{i(i-2n)-a(a+2n)}{(2a+2n+1)(a+n)}\right).
\end{aligned} \tag{81}$$

Thus,

$$P_3^{(d)} = aP^{(d)} - \frac{(2a+1)}{(2a+2n+1)(a+n)}\left[a(a+2n)(d-1)P^{(d)} + \sum_{i=0}^{d-1}\binom{2n}{i}\frac{B(a+i,a+2n-i)}{B(a,a)}i(2n-i)\right], \tag{82}$$

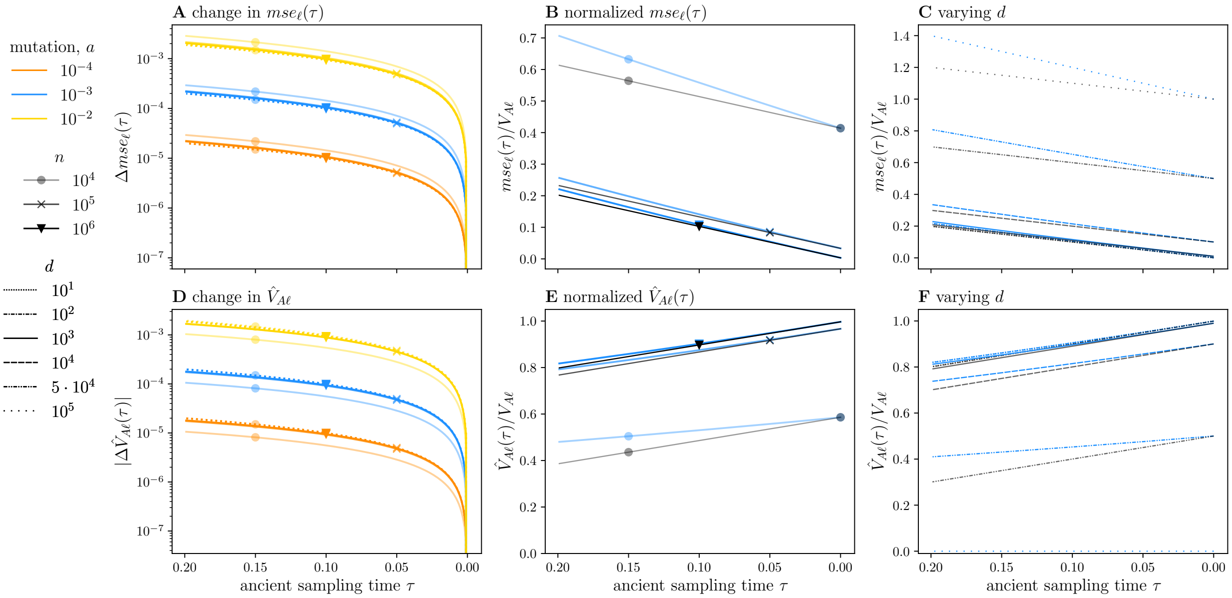

Fig S5. Approximations for the per locus contributions to the mean-squared error and estimated additive genetic variance across sample sizes, mutation rates, and detection thresholds. This plot is identical to Fig 2 in the main text except that we (i) include our approximations to the two statistics, Equation 15 in the main text and Equation 17 in the main text, and (ii) plot our results over a short time frame, $\tau \in [0.2, 0]$. In (A), the approximations are depicted as colored, dotted lines corresponding to each mutation rate and sample size pair. In (B), the approximations are denoted by the same markers and opacity as their blue counterparts. And, in (C), the approximations are provided in black, with line pattern indicating the threshold d.

And,

$$|P_3^{(d)} - aP^{(d)}| \approx \frac{a(d-1)}{n} P^{(d)} + \sum_{i=0}^{d-1} \binom{2n}{i} \frac{B(a+i, a+2n-i)}{B(a,a)} \left(\frac{i}{n} - \frac{i^2}{2n^2} \right) \tag{83}$$

where the approximation follows for $a \ll 1$ and $a \ll n$. Thus, $P_3^{(d_\ell)} \approx aP^{(d_\ell)}$ will be a very good approximation when $d \ll n$, but should also hold for modest d as long n is reasonably large. It is possible that when the mutational target is very large, e.g., $O(n)$, that approximation errors may be non-negligible for large enough d. However, large d implies a small β, thereby tempering any approximation errors in practice. An additional benefit of expressing $P_1^{(d)}$, $P_2^{(d)}$, and $P_3^{(d)}$ in terms of $P^{(d)}$ is that we can now take advantage of efficient coding of the beta-binomial probability mass function in the Python module `scipy` to compute analytical results for larger values of d.

Computations for large n and d. For large n, computing the terms in Equation 42 in S1 Text, excluding $P^{(d)}$ (which we compute using `scipy`), becomes computationally prohibitive. However, for large n, we can approximate these quantities as follows. Defining $z = \frac{d}{2n}$ and the incomplete

beta function as $I_z(x,y) = \frac{1}{B(x,y)}\int_0^z z^{x-1}(1-z)^{y-1}dz$,

$$\begin{aligned}
P_1^{(d)} &\approx \left(\frac{1}{n^2}\right) I_z(a+2,a) - \left(\frac{2}{n}\right) I_z(a+1,a) + P^{(d)} \\
P_2^{(d)} &\approx \frac{1}{a+n}\left(\frac{1}{n} I_z(a+2,a) - 2I_z(a+1,a) + nP^{(d)}\right) \\
P_3^{(d)} &\approx \frac{an(2n-1)}{(2a+2n+1)(a+n)} P^{(d)} + \left(\frac{2a+1}{(2a+2n+1)(a+n)}\right)\left(I_z(a+2,a) - 2I_z(a+1,a)\right).
\end{aligned} \tag{84}$$

These expressions allow us to compute the metrics for a much larger range of n and d values.

10 Polygenic score bias for recent genic selection

We provide evidence for the claim made in **Simulation results for recent directional selection** of the main text that, "$bias_\ell(\tau)$ will reach an equilibrium value that depends approximately on the asymmetry of the detection thresholds at the present day, which in turn, depends on both the timing and strength of selection". We treat the simplest case of a detection threshold $d = 1$, i.e., $\hat{\beta} = \beta$ if the locus is segregating in the GWA study sample. The time-varying distribution of the allele frequency is $f_t(\cdot)$, and necessarily depends on the timing and strength of selection. For $t > \tau_s$, the time of the onset of selection, $f_t(z) \propto z^{a-1}(1-z)^{a-1}$. For $t \leq \tau_s$, $f_t(z)$ will be skewed toward one and proportional to $\propto e^{\sigma z} z^{a-1}(1-z)^{a-1}$, where $\sigma = 4Ns$ is the population-scaled selection coefficient. For a larger τ_s, $f_t(z)$ for $t \leq \tau_s$ will have more time to shift toward the stationary distribution under selection.

From Equation 11 in the main text, we have that $bias_\ell(\tau)$, omitting the locus subscript is,

$$\begin{aligned}
bias(\tau) &= \mathbb{E}\left[(\bar{X} - X(\tau))(\beta - \hat{\beta})\right] \\
&= \beta\mathbb{E}\left[(\bar{X} - X(\tau))|\hat{\beta} = 0\right]\mathbb{P}\{\hat{\beta} = 0\} \\
&= \beta\left(\mathbb{E}\left[\bar{X}|\hat{\beta} = 0\right] - \mathbb{E}\left[X(\tau)|\hat{\beta} = 0\right]\right)\mathbb{P}\{\hat{\beta} = 0\} \\
&= \beta\left(-\mathbb{P}\{\bar{X} = -1|\hat{\beta} = 0\} + \mathbb{P}\{\bar{X} = +1|\hat{\beta} = 0\} - \mathbb{E}\left[X(\tau)|\hat{\beta} = 0\right]\right)\mathbb{P}\{\hat{\beta} = 0\}.
\end{aligned} \tag{85}$$

For large τ, $\mathbb{E}\left[X(\tau)|\hat{\beta} = 0\right] \to 0$, such that,

$$bias(\tau) \to \beta\left(\mathbb{P}\{\bar{X} = +1|\hat{\beta} = 0\} - \mathbb{P}\{\bar{X} = -1|\hat{\beta} = 0\}\right)\mathbb{P}\{\hat{\beta} = 0\}, \tag{86}$$

which shows that the $bias(\tau)$ will equilibrate at some value that depends on the difference between the $+$ and $-$ detection thresholds as well as the probability that $\hat{\beta} = 0$. This difference, in turn, depends on the time of the onset of selection and the selection coefficient (relative to the mutation rate) itself.

11 Fixation index and prediction accuracy

The complexity of human population history implies that an ancient sampling time of t years does not readily translate to a coalescent time of $\tau = t/2N$. As a result, it may be difficult to apply our theoretical results in practice. In lieu of an estimated ancient sampling time τ, we instead seek

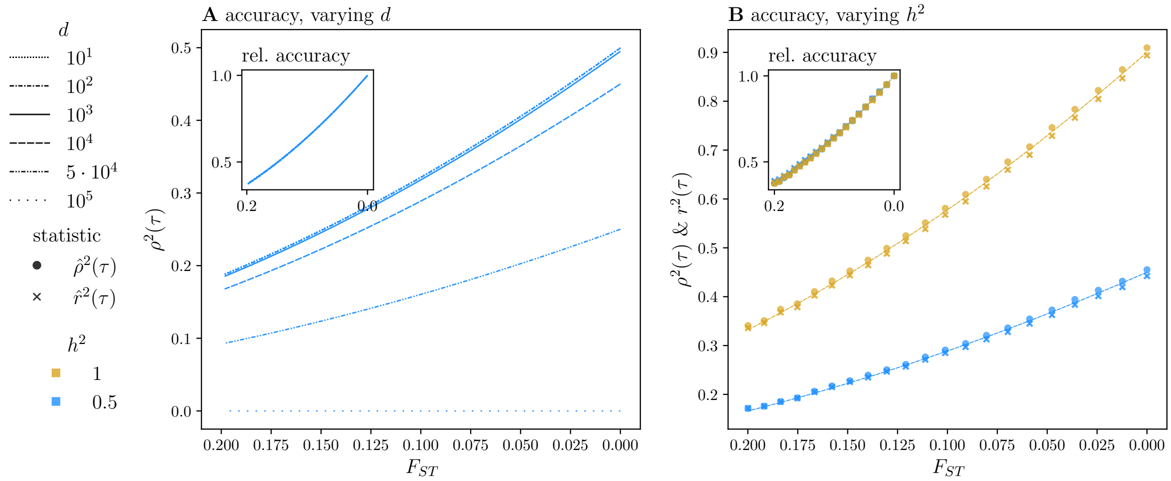

Fig S6. Polygenic score accuracy as a function of $\mathbf{F_{ST}}$. We reproduce Fig 3 in the main text with accuracy and relative accuracy as functions of F_{ST} instead of ancient sampling time τ.

to uncover the relationship between F_{ST} and our various metrics, which may, with some caveats, be more robust to demographic changes. And importantly, F_{ST} is readily measurable from ancient genotypic data.

F_{ST} is defined as the relative difference between within sample and across sample heterozygosity [21],

$$F_{ST} = \frac{\bar{z}(1-\bar{z}) - \overline{z(1-z)}}{\bar{z}(1-\bar{z})}, \tag{87}$$

where $\bar{z} = \frac{1}{2}[z(0) + z(\tau)]$ is the average of the allele frequencies in the contemporary and ancient populations, and $\overline{z(1-z)} = \frac{1}{2}[z(0)(1-z(0)) + z(\tau)(1-z(\tau))]$. We can approximate the expectation of F_{ST} by a ratio of expectations and solve under the assumptions of the recurrent mutation model and neutrality,

$$\mathbb{E}[F_{ST}] = \frac{\mathbb{E}[\bar{z}(1-\bar{z})] - \mathbb{E}[\overline{z(1-z)}]}{\mathbb{E}[\bar{z}(1-\bar{z})]} = \frac{\frac{a+1}{2(2a+1)} - \frac{1}{4}\left(1 + \frac{1}{2a+1}e^{-a\tau}\right)}{1 - \left[\frac{a+1}{2(2a+1)} + \frac{1}{4}\left(1 + \frac{1}{2a+1}e^{-a\tau}\right)\right]}. \tag{88}$$

We include derivations of the constituent expectations for completeness,

$$\begin{aligned}\mathbb{E}[\bar{z}(1-\bar{z})] &= \frac{1}{2}\mathbb{E}[z(0) + z(\tau)] - \frac{1}{2}\mathbb{E}[(z(0) + z(\tau))^2] \\ &= \frac{1}{2} - \frac{1}{2}\left(\frac{a+1}{2(2a+1)} + \frac{1}{4} + e^{-a\tau}\frac{1}{4(2a+1)}\right).\end{aligned} \tag{89}$$

And,

$$\begin{aligned}\mathbb{E}[\overline{z(1-z)}] &= \frac{1}{2}\mathbb{E}[z(0)(1-z(0)) + z(\tau)(1-z(\tau))] \\ &= \frac{1}{2} - \frac{a+1}{2(2a+1)}.\end{aligned} \tag{90}$$

Using the results from Equation 88 in S1 Text, in Fig S6 in S1 Text, we reproduce Fig 3 in the main text with an x-axis of pairwise F_{ST} between the focal population and the GWA study population instead of ancient sampling time.

12 Comparison to the results of Wang et al. 2020

In *Polygenic score accuracy* of **Analytical Results** in the main text, we found that relative accuracy was fairly insensitive to many of the model parameters. This allows us to more readily compare our theoretical results with those of Wang et al. 2020, who also generated predictions for accuracy decay in out-of-sample predictions in humans. In Fig S7 in S1 Text, we plot our neutral theory (n = 350,000, d = 1,000, and $a = 10^{-3}$) as a function of pairwise divergence,as measured by F_{ST} (see Section 11 in S1 Text). Alongside, we plot Wang et al.'s predictions for accuracy reductions in individuals of South Asian (sas), East Asian (eas), and African (afr) ancestries in the UK Biobank relative to a sample of individuals of European (eur) ancestry as a function of observed F_{ST} with eur. In addition, we plot the reductions in accuracy in each ancestry group that were observed in the data set.

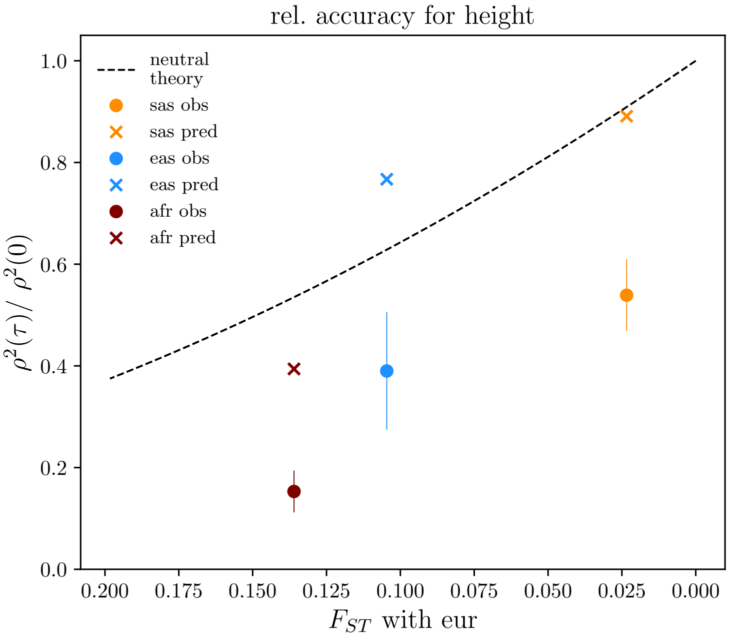

Fig S7. Relative polygenic score accuracy. We compare our theoretical results (dashed line; $n = 350,000$, $d = 1000$, and $a = 10^{-3}$) for relative accuracy reductions to those of Wang et al. [7] for height in individuals of non-European ancestry. Each ancestry group, South Asian (sas), East Asian (eas), and African (afr) is distinguished by color. The x's demarcate Wang et al.'s predictions, while the circles denote the observed accuracy reductions; error bars are 95% confidence intervals. Theoretical values of F_{ST} are computed according to Equation 88 in S1 Text; observed values for each ancestry group are from [7].

In contrast to our theory, Wang et al. take into account the combined effects of (observed) differences in LD and allele frequencies between ancestry groups. Thus, since our theory only accounts for allele frequency changes, it is not surprising that we underestimate the accuracy reductions observed in individuals of African ancestry. Surprisingly, our predictions exceed or approximate those of Wang et al. for sas and eas ancestries—which all underestimate the observed accuracy reductions.

Altogether, these results suggest that accurately predicting out-of-sample accuracy reductions will require more complex modeling of the underlying demographic processes and environmental factors contributing to phenotypic variation. In short, the relationship between F_{ST} and prediction accuracy is not simple.

13 Necessary moments, under neutrality and at stationarity

We provide analytic expressions for the moments which constitute the various metrics under the assumption of equal mutation rates. In addition, we provide simplified expressions when the detection thresholds are equal.

Moments of the population allele frequency and genotype. Because the population is at stationarity, these moments are time-invariant. They require integration over the stationary density of the population allele frequency, which is beta-distributed, and in (b) require integration over the Hardy-Weinberg sampling process.

a. *Moments of the population allele frequency:*

$\mathbb{E}[Z_\ell] = \frac{1}{2}$, $\mathbb{E}\left[Z_\ell^2\right] = \frac{a+1}{2(2a+1)}$, and $\mathbb{E}[Z_\ell(1-Z_\ell)] = \frac{a}{2(2a+1)}$.

b. *Moments of a genotype:*

$\mathbb{E}[X_{i\ell}] = 0$ and thus $\mathbb{V}[X_{i\ell}(t)] = \mathbb{E}\left[X_{i\ell}^2(t)\right] = \frac{a+1}{2a+1}$.

Moments specific to the GWA study. These moments require integration over the stationary density of the population allele frequency and the sampling probabilities for a sample of n individuals.

a. *Moments of the mean genotype in the GWA study sample:*

$\mathbb{E}\left[\bar{X}_\ell\right] = 0$ and $\mathbb{E}\left[\bar{X}_\ell^2\right] = \frac{1}{n}\left(\frac{a+n}{2a+1}\right)$.

b. *Product of the mean genotype in the GWA study sample and the effect estimate:*

$$\begin{aligned}\mathbb{E}\left[\bar{X}_\ell\hat{\beta}_\ell\right] = \mathbb{E}\left[\mathbb{E}\left[\bar{X}_\ell\hat{\beta}_\ell|\bar{X}_\ell\right]\right] &= \beta_\ell\mathbb{E}\left[\bar{X}_\ell\mathbb{1}_{\{\bar{X}_\ell\in(\gamma-1,1-\gamma)\}}\right]\\ &= \beta_\ell\sum_{i=d_{\ell 1}}^{2n-d_{\ell 2}}\left(\frac{i-n}{n}\right)\binom{2n}{i}\mathbb{E}\left[Z_\ell^i\left(1-Z_\ell\right)^{2n-i}\right]\\ &= \beta_\ell\sum_{i=d_{\ell 1}}^{2n-d_{\ell 2}}\left(\frac{i-n}{n}\right)\binom{2n}{i}\frac{B(a+i,b+2n-i)}{B(a,b)}.\end{aligned} \tag{91}$$

And, for $d_{\ell 1} = d_{\ell 2}$,

$$\mathbb{E}\left[\bar{X}_\ell\hat{\beta}_\ell\right] = 0. \tag{92}$$

And,

$$\mathbb{E}\left[\bar{X}_\ell^2\hat{\beta}_\ell\right] = 2\beta_\ell\sum_{i=d_{\ell 1}}^{n-d_{\ell 2}}\left(\frac{i-n}{n}\right)^2\binom{2n}{i}\frac{B(a+i,a+2n-i)}{B(a,a)} \tag{93}$$

For $d_{\ell 1} = d_{\ell 2} = d_\ell$,

$$\begin{aligned}\mathbb{E}\left[\bar{X}_\ell^2\hat{\beta}_\ell\right] &= \beta_\ell\left(\frac{a+n}{n(2a+1)} - 2\sum_{i=0}^{d_\ell-1}\left(\frac{i-n}{n}\right)^2\binom{2n}{i}\frac{B(a+i,a+2n-i)}{B(a,a)}\right)\\ &= \beta_\ell\left(\frac{a+n}{n(2a+1)} - 2P_1^{(d_\ell)}\right).\end{aligned} \tag{94}$$

Under our simple threshold model, the corresponding second moment of $\hat{\beta}_\ell$ is equal to the previous expression multiplied by β_ℓ.

c. *First moment of the mean phenotype in the GWA study sample:* $\mathbb{E}\left[\bar{Y}\right] = 0$.

d. *First moment of the estimated intercept term:*

$$\mathbb{E}\left[\hat{C}\right] = \mathbb{E}\left[\bar{Y}\right] - \sum_{\ell=1}^{L} \mathbb{E}\left[\bar{X}_\ell \hat{\beta}_\ell\right] = C - \sum_{\ell=1}^{L} \beta_\ell \sum_{i=d_{\ell 1}}^{2n-d_{\ell 2}} \left(\frac{i-n}{n}\right) \binom{2n}{i} \frac{B(a+i, a+2n-i)}{B(a,a)}, \tag{95}$$

which, for equal detection thresholds equals 0.

Moments involving both the ancient and contemporary genotypes. These moments involve quantities from two time points: the ancient sampling time τ and the GWA study at the present. To compute these moments, we use the spectral representation of the *tdf* (Section 6 in S1 Text).

a. *Product of the first moments of the ancient and contemporary mean genotype:*

$$\begin{aligned}
\mathbb{E}\left[X_\ell(\tau)\bar{X}_\ell(0)\right] &= \frac{1}{n}\sum_{i=1}^{n} \mathbb{E}\left[\mathbb{E}\left[X_\ell(\tau)X_{i\ell}(0) | Z_\ell(0), Z_\ell(\tau)\right]\right] \\
&= \mathbb{E}\left[(2Z_\ell - 1)(2Z_\ell(\tau) - 1)\right] \\
&= \frac{1}{B(a,b)} \sum_{k=0}^{1} \frac{e^{-\lambda_k \tau}}{\langle B_k, B_k\rangle_\pi} \langle 2z-1, B_k\rangle_\pi^2 \\
&= e^{-a\tau}\left(\frac{1}{2a+1}\right).
\end{aligned} \tag{96}$$

b. *Product of the moments of the ancient and contemporary mean genotypes, and the effect estimate:*

$$\begin{aligned}
\mathbb{E}\left[\bar{X}_\ell \hat{\beta}_\ell X_\ell(\tau)\right] &= \sum_{i=d_{\ell 1}}^{2n-d_{\ell 2}} \left(\frac{i-n}{n}\right) \binom{2n}{i} \sum_{k=0}^{1} \frac{e^{-\lambda_k \tau}}{\langle B_k, B_k\rangle_\pi} \langle 2z-1, B_k\rangle_\pi \langle z^i (1-z)^{2n-i}, B_k\rangle_\pi \\
&= e^{-a\tau}\beta_\ell \sum_{i=d_\ell}^{2n-d_\ell} \left(\frac{(i-n)^2}{n(a+n)}\right) \binom{2n}{i} \frac{B(a+i, a+2n-i)}{B(a,a)}.
\end{aligned} \tag{97}$$

For equal detection thresholds, d_ℓ, and using the variables defined in Equation 42 in S1 Text, we have,

$$\mathbb{E}\left[\bar{X}_\ell \hat{\beta}_\ell X_\ell(\tau)\right] = \beta_\ell e^{-a\tau}\left(\frac{1}{2a+1} - 2P_2^{(d_\ell)}\right). \tag{98}$$

The corresponding second moment of $\hat{\beta}_\ell$ is equal to the previous expression multiplied by β_ℓ.

Moments involving the ancient (but not contemporary) genotype. These moments involve the ancient genotype $X_\ell(\tau)$ and the contemporary effect estimate $\hat{\beta}_\ell$, but not the contemporary genotypes.

a. *Product of the first moments of the ancient genotype and the effect estimate:*

$$\begin{aligned}
\mathbb{E}\left[X_\ell(\tau)\hat{\beta}_\ell\right] &= \frac{1}{B(a,a)} \sum_{i=d_{\ell 1}}^{2n-d_{\ell 2}} \binom{2n}{i} \sum_{k=0}^{1} \frac{e^{-\lambda_k \tau}}{\langle B_k, B_k\rangle_\pi} \langle z^i(1-z)^{2n-i}, B_k\rangle_\pi \langle 2z-1, B_k\rangle_\pi \\
&= e^{-a\tau} \sum_{i=d_{\ell 1}}^{2n-d_{\ell 2}} \binom{2n}{i} \left(\frac{B(a+i, a+2n-i)}{B(a,a)}\right) \left(\frac{i-n}{a+n}\right) = 0,
\end{aligned} \tag{99}$$

for equal thresholds $d_{\ell 1} = d_{\ell 2}$. This result is due to the fact that, in Equation 99 in S1 Text, the i-th term is equal to the $(2n-i)$-th term (and the n-th term is 0).

b. *Product of the second moments of the ancient genotype and first moment of the effect estimate:*

$$\begin{aligned}\mathbb{E}\left[X_\ell^2(\tau)\hat{\beta}_\ell\right] &= \frac{\beta_\ell}{B(a,b)} \sum_{i=d}^{2n-d} \binom{2n}{i} \sum_{k=0}^{2} \frac{e^{-\lambda_k \tau}}{\langle B_k, B_k\rangle_\pi} \langle 1-2z+2z^2, B_k\rangle_\pi \langle z^i(1-z)^{2n-i}, B_k\rangle_\pi \\ &= \frac{\beta_\ell}{2a+1} \sum_{i=d}^{2n-d} \binom{2n}{i} \frac{B(a+i, a+2n-i)}{B(a,a)} \\ &\quad \times \left[(a+1) + e^{-(2a+1)\tau}\left(\frac{(2a+1)i(i-2n)+an(2n-1)}{(2a+2n+1)(a+n)}\right)\right].\end{aligned} \tag{100}$$

For equal detection thresholds d_ℓ, and Using the terms defined in Equation 42 in S1 Text, we can express Equation 100 in S1 Text more succinctly,

$$\mathbb{E}\left[X_\ell^2(\tau)\hat{\beta}_\ell\right] = \frac{\beta_\ell}{2a+1}\left[(a+1)(1-2P^{(d_\ell)}) - 2e^{-(2a+1)\tau}P_3^{(d_\ell)}\right]. \tag{101}$$

The moment $\mathbb{E}\left[X_\ell^2(\tau)\hat{\beta}_\ell^2\right]$ is equal to the previous expression multiplied by β_ℓ.

References

1. Simons YB, Bullaughey K, Hudson RR, Sella G. A population genetic interpretation of GWAS findings for human quantitative traits. PLoS Biology. 2018;16. https://doi.org/10.1371/journal.pbio.2002985.

2. Sham PC, Purcell SM. Statistical power and significance testing in large-scale genetic studies. Nature Reviews Genetics. 2014;15(5):335–346. https://doi.org/10.1038/nrg3706.

3. Meuwissen TH, Hayes BJ, Goddard ME. Prediction of total genetic value using genome-wide dense marker maps. Genetics. 2001;157(4):1819–1829. https://doi.org/10.1093/genetics/157.4.1819.

4. de los Campos G, Vazquez AI, Fernando R, Klimentidis YC, Sorensen D. Prediction of Complex Human Traits Using the Genomic Best Linear Unbiased Predictor. PLoS Genetics. 2013;9(7). https://doi.org/10.1371/journal.pgen.1003608.

5. Barton NH, Etheridge AM, Véber A. The infinitesimal model: Definition, derivation, and implications. Theoretical Population Biology. 2017;118:50–73. https://doi.org/https://doi.org/10.1016/j.tpb.2017.06.001.

6. Vilhjálmsson BJ, Yang J, Finucane HK, Gusev A, Lindström S, Ripke S, et al. Modeling Linkage Disequilibrium Increases Accuracy of Polygenic Risk Scores. American Journal of Human Genetics. 2015;97(4):576–592. https://doi.org/10.1016/j.ajhg.2015.09.001.

7. Wang Y, Guo J, Ni G, Yang J, Visscher PM, Yengo L. Theoretical and empirical quantification of the accuracy of polygenic scores in ancestry divergent populations. Nature Communications. 2020;11(1). https://doi.org/10.1038/s41467-020-17719-y.

8. Griffiths RC, Spano D. Diffusion processes and coalescent trees. arXiv. 2010. http://arxiv.org/abs/1003.4650.

9. Song YS, Steinrücken M. A simple method for finding explicit analytic transition densities of diffusion processes with general diploid selection. Genetics. 2012;190(3):1117–1129. https://doi.org/10.1534/genetics.111.136929.

10. Nielsen R, Akey JM, Jakobsson M, Pritchard JK, Tishkoff S, Willerslev E. Tracing the peopling of the world through genomics. Nature. 2017;541(7637):302–310.

11. Gazal S, Finucane HK, Furlotte NA, Loh PR, Palamara PF, Liu X, et al. Linkage disequilibrium-dependent architecture of human complex traits shows action of negative selection. Nature Genetics. 2017;49(10):1421–1427. https://doi.org/10.1038/ng.3954.

12. O'Connor LJ, Schoech AP, Hormozdiari F, Gazal S, Patterson N, Price AL. Extreme Polygenicity of Complex Traits Is Explained by Negative Selection. American Journal of Human Genetics. 2019;105(3):456–476. https://doi.org/10.1016/j.ajhg.2019.07.003.

13. Zeng J, Xue A, Jiang L, Lloyd-Jones LR, Wu Y, Wang H, et al. Widespread signatures of natural selection across human complex traits and functional genomic categories. Nature Communications. 2021;12(1). https://doi.org/10.1038/s41467-021-21446-3.

14. Sohail M, Maier RM, Ganna A, Bloemendal A, Martin AR, Turchin MC, et al. Polygenic adaptation on height is overestimated due to uncorrected stratification in genome-wide association studies. eLife. 2019;8:1–17. https://doi.org/10.7554/elife.39702.

15. Berg JJ, Harpak A, Sinnott-Armstrong N, Joergensen AM, Mostafavi H, Field Y, et al. Reduced signal for polygenic adaptation of height in UK Biobank. eLife. 2019;8. https://doi.org/10.7554/elife.39725.

16. Zhou X, Carbonetto P, Stephens M. Polygenic Modeling with Bayesian Sparse Linear Mixed Models. PLoS Genetics. 2013;9(2):e1003264. https://doi.org/10.1371/journal.pgen.1003264.

17. Moser G, Lee SH, Hayes BJ, Goddard ME, Wray NR, Visscher PM. Simultaneous Discovery, Estimation and Prediction Analysis of Complex Traits Using a Bayesian Mixture Model. PLoS Genetics. 2015;11(4):e1004969. https://doi.org/10.1371/journal.pgen.1004969.

18. Zhang Y, Qi G, Park JH, Chatterjee N. Estimation of complex effect-size distributions using summary-level statistics from genome-wide association studies across 32 complex traits. Nature Genetics. 2018;50(9):1318–1326. https://doi.org/10.1038/s41588-018-0193-x.

19. Hormozdiari F, Gazal S, Van De Geijn B, Finucane HK, Ju CJT, Loh PR, et al. Leveraging molecular quantitative trait loci to understand the genetic architecture of diseases and complex traits. Nature Genetics. 2018;50(7):1041–1047. https://doi.org/10.1038/s41588-018-0148-2.

20. Zeng J, De Vlaming R, Wu Y, Robinson MR, Lloyd-Jones LR, Yengo L, et al. Signatures of negative selection in the genetic architecture of human complex traits. Nature Genetics. 2018;50(5):746–753. https://doi.org/10.1038/s41588-018-0101-4.

21. Weir BS. Genetic Data Analysis II. Sunderland, MA: Sinauer Associates, Inc.; 1996.
