# Supplementary material for: Polygenic score accuracy in ancient samples: Quantifying the effects of allelic turnover
Source: PLoS Genet. 2022 May 6;18(5):e1010170. doi: 10.1371/journal.pgen.1010170 (PMC9116686; doi:10.1371/journal.pgen.1010170)
Supplement: Attachment — Submitted filename: response_to_reviewers.pdf [file pgen.1010170.s003.pdf]

Dear Drs. Lohmueller, Payseur, and reviewers, please find our detailed responses to the comments below, indicated in blue.

Dear Dr Steinrücken,

[...]

Please let us know if you have any questions while making these revisions.

Yours sincerely,

Kirk E Lohmueller
Guest Editor
PLOS Genetics

Bret Payseur
Section Editor: Evolution
PLOS Genetics

Thank you for submitting this work to PLOS Genetics. It has been evaluated by 3 reviewers. All generally liked the manuscript. The reviewers provide a number of comments to improve the manuscript. None stood out as being more critical than others. I think addressing these comments, even those that were presented as suggestions/not required by the reviewers, in your revision will improve the readability and applicability of your work.

Thank you very much for the feedback on our manuscript. Below, we have provided responses to the reviewer comments (in blue), including references to the corresponding changes in the manuscript. The changes in the marked up manuscript are indicated in red and line numbers refer to the marked up version of the manuscript. Where suggested, we have clarified portions of the main text. In particular, we have expanded the introduction and discussion to address, for example, the relationship of our work to similar studies (Wang et al. 2020 and Yair and Coop 2021).

In addition, we have added several new sections to the Supplementary Information to (1) relate our simple threshold model to best linear unbiased prediction (S1.4.2); (2) compute prediction accuracy under a parameterization consistent with the UK Biobank and using detection thresholds derived from UK Biobank summary statistics (S1.8); (3) relate divergence as measured by Fst and prediction accuracy (S1.11); and (4) compare our predictions with those of Wang et al. 2020 (S1.12). No other substantial changes were made to the supplement.

We have also made the following minor changes:

a) We made some small stylistic changes to the Author Summary.
b) We removed an erroneous beta hat in the denominator of the first expression in Equation (18).
c) Line 489 should have referenced Equation (19), not Equation (20) as previously. This is now fixed.
d) We had accidentally omitted a section header when combining the supplementary materials. There is now a section Supporting Information S1.7 that includes detailed derivations of the metrics with subsections (formerly sections) for each statistic.

Reviewer's Responses to Questions

Comments to the Authors:
Please note here if the review is uploaded as an attachment.

Reviewer #1: This manuscript by Carlson and colleagues considers the effect of "allelic turnover" on polygenic scores fit to ancient samples. "Allelic turnover," as the authors define it, is relevant to the problem of polygenic score portability in part because causal variants that explain trait variation in an ancient sample may experience frequency changes that make them undetectable in modern GWAS. The authors use a diffusion approach to study several summaries of polygenic score performance. I have not checked all the equations, but the work appears to be well done. I have a few comments, which I list not as requirements for publication (which I leave up to the editor), but rather as friendly reactions and suggestions.

1) The model here assumes that the ancient sample is from a population directly ancestral to a contemporary population, when in fact many ancient samples may not fit this description straightforwardly. (See e.g. Schraiber 2018, PMID: 29167200). I think it is totally fair for the authors to model things the way they do, as it captures a lot of the intuition and allows them just to think about one forward diffusion. However, the paper might benefit from a clearer discussion of this potential caveat.

Indeed, in practice, ancient samples may not be sampled from a population directly ancestral to that of the GWA study. If this is the case, we can think of the ancient individual as derived from a population that split from the GWA study population at some point in the past. Thus, as we show in Supporting Information S1.1 and discuss in Models and metrics (lines 151-160), this scenario is already encompassed by our model: the sampling time is merely increased by the divergence time between the two populations.

To make this more explicit in the main text, we have:

1) Directly addressed this point in the introduction by adding (lines 96-101): “The latter scenario can represent an ancient individual being sampled from a population not

directly ancestral to that of the GWA study as the two populations must have diverged at some point in the past. This scenario may, to a first approximation, describe population displacement events, thought to be ubiquitous in the history of humans (e.g.\ \cite{Liu2021}). However, human history is additionally characterized by numerous admixture events and population size changes (e.g.\ \cite{Liu2021}) which are not yet captured within our modeling framework."

2) More explicitly emphasized the population split scenario in the main text (see our response to (2) directly below).

2) Along similar lines to (1), I was struck by how much of the framing is in terms of ancient samples when a lot of the intuitions developed are relevant to pairs of contemporary populations. The model is more straightforwardly related to ancient populations (though see (1)), but I think it'd be interesting for the authors to give more space to thinking about the relevance for contemporary populations. There is a paragraph on this in the discussion, but only a hint of a quantitative comparison is given, and as far as I can tell mainly for empirical results. It would be useful to include a comparison with the modeling approach of Wang, Guo et al (ref 16).

We have now more explicitly addressed the population split scenario, which approximates the relationship between contemporary populations, in the main text as follows:

a) We moved Supplementary Figure S1 to the main text, now Figure 1a and 1b.
b) The first paragraph of the Model and Metrics section includes more in depth discussion of the split scenario (lines 151-160), previously provided in Supporting Information S1.1.

In regards to a comparison to the results of Wang et al. 2020:

a) We have now added a more detailed comparison of the model used in Wang et al. to the introduction. See lines 83-88: "Previous quantitative genetic approaches…"
b) And, we provide a more quantitative comparison in Supporting Information S1.12, referenced in the discussion in lines 668-686.

3) arXiv:1909.00892 has a nice discussion of the relevance of allelic turnover to polygenic score comparisons as proxies for phenotype comparisons. This is captured in the various summaries studied, but I would have liked more emphasis on distinguishing within-population accuracy vs. getting changes in the mean over time correct in the discussion.

We had already implicitly addressed the issue of within-population accuracy vs. changes in the mean over time, but we have made several changes to make our treatment more explicit:

(a) We added the following to Section 3.1 on the bias (Lines 334-337) : "It follows that in the absence of additional perturbing forces, an estimate of the mean polygenic score from a sample of n_a ancient individuals will also be unbiased, and therefore, will on average accurately reflect the lack of change in the mean phenotype."
(b) We added text to Section 3.4 (Polygenic score accuracy, lines 510-513): "As with the preceding statistics, when $\tau=0$, $\rho^(\tau)$ approximates the accuracy of the

polygenic score \emph{within} the GWA study population. Relative accuracy directly measures reductions in accuracy relative to the GWA study population."

(c) We commented on the implications of our non-zero bias result for detection of mean differences between ancient polygenic scores in the Discussion, lines 664-670: "The effects of selection on the bias have implications…At the same time..."

4) A new preprint by Yair & Coop is relevant for this study and takes a complementary modeling approach https://doi.org/10.1101/2021.09.10.459833 . (I know what this is starting to look like, and I swear I'm not Graham Coop.)

We have now added the following text to the Discussion, lines 610-617:

"In addition, increasing the detection threshold yielded lower polygenic score accuracy, as a SNP was less likely to have a non-zero effect. These results are broadly consistent with a concurrent study by Yair and Coop \cite{Yair2021}, in which they used simulations to assess cross-population prediction accuracy, defined as the ratio of the variance of and individual's polygenic score to that of their genetic value, under neutrality and in the presence of stabilizing selection. When Yair and Coop restricted the polygenic score to the top one percent of SNPs, roughly analogous to altering the detection threshold, they similarly found that the accuracy declined in the focal population."

5) I found the explanations of the results in the main text to be, in some places, a little wordy and hard to follow. I think it is worthwhile to communicate the parts of the intuition that have to do with allele frequencies changing into or out of the GWAS-detectable range, but I got lost in some of the verbal explanations. I also thought the summary in the intro was in too much depth for not having seen the model yet. The repeated structure of the results subsections, though helpful for comprehension, also get to feel redundant. I would urge the authors to cut down the main text, lean more on their figures, and even possibly make use of tables to summarize some of the main results (e.g. in the intro).

In the course of revision, we have made numerous clarifying changes to the paper. We, however, have not substantially restructured the main text in line with the latter part of this comment.

6) I appreciated that the authors brought in literature on genetic prediction outside humans.

7) The final paragraph is perhaps missing a mention of environmental differences and GxE.

We have added additional discussion of changes in environment and GxE to the final paragraph, lines 714-717: "Indeed, differences in causal effects across contemporary populations**, perhaps due to changes in the environment, epistasis, or gene-by-environment interactions**, likely contribute to accuracy reductions...Indeed, Cox et al..."

Minor comments

a) line 139, I assume that N is a diploid population size because time units are scaled in 2N generations, but please specify.

We have now clarified (in lines 154-155) that the diploid population size is 2N: “An ancient sampling time of \tau corresponds to 2N \cdot \tau generations in the past, with 2N as the diploid population size.”

b) line 167, is it meant that this quantity is often called a "genetic value" specifically in this paper? I have not seen it much in other places. If it's meant that it's a general term, please provide a citation.

We have now cited Meuwissen et al. 2001 (in what is now line 186).

c) p. 5, I might have missed it, but I don't see it explicitly stated here that eq. 2 assumes linkage equilibrium as well. (It shows up in the beginning of 2.3 but should probably be here first.)

Thank you for finding this error. The assumption of linkage equilibrium now precedes equation 2: “Assuming linkage equilibrium between loci as well as…” (lines 194-5).

Reviewer #2: In this manuscript, Carlson et al. provide a masterful assessment of polygenic score accuracy decreases due to allelic turnover, in the context of ancient populations that are ancestral to the GWAS panel on which a phenotype was measured. They quantify this decrease as a function of various parameters of interest, including the temporal distance between the ancient and present-day population, the power to detect significantly-associated loci, and trait heritability. They also produce simulations to determine how much weak selection on trait-associated variants biases the quantities of interest.

The manuscript is well written and it is clear that the authors have done a great deal of work to reach their conclusions, and evaluate various quantities of interest for geneticists to assess how reliable a score for an ancient individual would be. I only have very minimal suggestions for changes or additions to the manuscript, that I don't deem crucial for the manuscript to be accepted for publication:

To provide a clearer picture for researchers choosing to do (or not do) polygenic prediction on ancient samples, it would be nice to work with values from a concrete phenotype and GWAS study: given the size and heritability estimates from the latest UK Biobank GWAS or Biobank Japan on a particular phenotype (e.g. height), how much would one mis-estimate this phenotype in a population that is X years away from UKBB or BBJ, in the past, solely due to allelic turnover?

We have now included a supplementary section (Supporting Information S1.8) in which we compute prediction accuracy under a parameterization consistent with the UK Biobank. In short,

we use the summary statistics from the Price lab's analysis of the UK Biobank data on "body height" to specify the relationship between minimum effect size and the detection threshold. Then, we assume several plausible effect size distributions, including a distribution estimated from GWA study summary statistics in Zheng et al. 2018, and with these distributions and the empirically derived thresholds compute the approximate sample correlation coefficient for arbitrary sampling time. This section is referenced in the main text in lines 623-625.

Rarely are polygenic scores in the ancient DNA literature computed for populations that are directly ancestral to the present-day GWAS population, as there has been substantial population turnover and admixture in human populations during the Holocene and Pleistocene. It might be worth emphasizing this more strong in the Discussion so that the reader is aware that a population sampled X years in the past (in the same location as a present-day population) does not necessarily correspond to your scenario of an ancestral population that is separated by X years from the present-day GWAS populations.

We have now explicitly addressed this alongside the other limitations of our model in the Discussion. Lines 687-690: "For one, our model does not incorporate the complex demographic processes, such as admixture and population size changes, inherent in human history. This implies that an ancient sampling time of t years in the past likely does not correspond to a sampling time of $\tau = t/2N$ in our model, where $2N$ is the contemporary population size."

Please also see our responses to comments (1) and (2) from Reviewer #1.

In this vein, the manuscript would benefit from moving some of the results from Supplementary Text S1.1 to the main text (at a minimum the figure and the final conclusion), as those would more generally applicable to the larger set of researchers not necessarily working with directly ancestral populations, or working with diverged populations from the GWAS one, outside the ancient genomic literature.

We have followed the suggestion and included more details in the main text! Please see our responses to reviewer #1's points (1) and (2) for details.

Following up on this, could the authors also express the turnover-caused decreased in accuracy in terms of Fst values, rather than the ancient sampling time in coalescent units? This would make it a lot easier for an empirical researcher to assess how much of a decrease in accuracy due to allelic turnover they would expect for their ancient (or out-of-space) polygenic predictions, given that Fst values between present-day and ancient populations are readily obtainable (assuming stationarity and that allelic turnover is the only contributor to the decrease in accuracy).

We have now added a supplementary section (Supporting Information S1.11) that:

(a) Computes Fst under the recurrent mutation model as a function of ancient sampling time (or population divergence).

(b) Plots accuracy and relative accuracy against Fst, analogous to the plots in the main text (Figure S6).
(c) And discusses the caveat that complex population history may not yield a simple relationship between Fst and divergence time or prediction accuracy.

This section is cited in the Introduction in lines 106-108, in Analytical Results in lines 315-317 and lines 515-16.

In addition, in Supporting Information S1.12, we also compare our theory to results from Wang et al. 2020 with respect to Fst. In particular, see Figure S7.

I would be curious to see how accuracy decreases with increasing the number of ancient individuals used in the computation of polygenic score, e.g. how much more / less problematic is it to compute the mean polygenic score for a population of ancient hunter-gatherers vs. computing a polygenic score individually for each ancient hunter-gatherer in the population. How is the variance for these different estimates affected by the sampling time?

In Section 3.4, we showed that the expectation of the sample correlation coefficient, i.e. polygenic score accuracy, is not influenced by the ancient sample size, see Equation (19). The estimated additive genetic variance, however, is influenced by the ancient sample size in the form of a constant factor, see Equation (16). Hence the sample size factor in Equation (19), which relates the estimated additive genetic variance to the approximate sample correlation coefficient.

If one were to ask 'What are the statistical properties of an estimate of the mean polygenic score in a population?' and 'How does the variance depend on the sample size? the ancient sampling time?', we would answer:

First, such an estimator is necessarily unbiased under neutrality and our other assumptions (now emphasized in lines 334-337). However, computing the variance of such an estimator would require extension of the theoretical derivation here, and we thus believe that treating this question with the appropriate care is outside the scope of the present study.. We suspect that more ancient samples will reduce the variance of the estimator.

Beyond recent directional selection, could the authors provide some intuition as to how persistent negative selection would affect their results? i.e. How would these be affected when the magnitude of the effect size estimates determines the probability that the individual variants will be removed from the population?

We address the two questions as follows:

(a) Negative selection can easily be captured by our model: one simply reverses which allele is designated as positive or negative. We have now added text to emphasize this in Section 4 (*Simulation*) in lines 550-553: "If we instead designated the A_1 allele as the beneficial allele, ..."

(b) We have clarified that under directional selection, the selection coefficient is proportional to the locus effect size, lines 557-559: “Under directional selection, σ is proportional to the locus effect size β; mutations with larger effect sizes will be more likely to establish and achieve appreciable frequencies (Chevin 2008).”

Reviewer #3: In this manuscript, Maryn et al. provide theoretical results for the effects of mutation and drift on the performance of polygenic scores estimated from a modern population. They focus on the performance of these scores in an ancestral population (from which we might have archaeological samples), and they note that the same calculations are applicable to different contemporaneous populations (at least under a simple demography).

I quite liked the paper. Most of my initial concerns were addressed in the SI. I have one major comment related to the PRS approximation, and some more minor comments more related to presentation.

1. The authors use an approximation for the behavior of a PRS: at some threshold that depends on the sample allele frequency and the true effect size, the effect of that SNP is observed (with no estimation error), and at any lower threshold it is unobserved. This approximation was previously used by Simons et al 2018, and it is clearly a useful one, as they justify in SI.

The authors state that their approximations are insensitive to the true genetic architecture (distribution of beta), but I don’t quite agree. The threshold model is aligned with the “pruning + thresholding” PRS method, and this method can be viewed as an approximate shrinkage/Bayesian posterior approach. In particular it is justified under sparse genetic architectures: only a small proportion of SNPs have a true nonzero effect, so for any SNP that you can’t tell if it’s zero, it is probably zero and you should not put it in the score.

Under an infinitesimal genetic architecture, a totally different approach is called for, using a Gaussian prior. The resulting posterior mean/MAP estimator is variously known as BLUP, ridge regression or LDpred-inf. With this approach, the PRS weights are simply
$\hat{\beta} = c X^T Y$
for some c depending on the sample size, the sample allele frequency, and the prior effect-size variance (but not depending on Y or β). I expect that this approach would behave totally differently from the threshold model: every SNP has the same estimation error in per-normalized-genotype units, there is no bias, and the total amount of error depends critically on the number of segregating sites.

I think that state-of-the-art PRS methods can be viewed heuristically as doing something in between BLUP and pruning + thresholding.

To finally state a question, will there be a qualitative difference in the performance of polygenic scores for an extremely polygenic trait coupled with a BLUP estimator?

We agree that we had not sufficiently addressed the relationship between our threshold model and other prediction models, such as BLUP. We have now expanded Supporting Information S1.4 to address the questions/comments questions that you raise. In short, in Supporting Information S1.4.2, we provide a heuristic argument that the threshold model can be viewed as an approximation to the BLUP model. In addition, we note that centering and scaling the genotypes and phenotypes simplifies our argument but is not necessary. (In Section S1.5 we more formally discuss the effects of centering and scaling on our statistics. In short, standardization is statistically convenient but cumbersome when modeling the evolution of allele frequencies, and does not alter our conclusions.)

We reference Supporting Information S1.4 in the Introduction in line 81, and more explicitly in lines 213-214, “In Supporting Information S1.4, we relate Equation (3) to two alternative estimation procedures: maximum likelihood estimation (MLE) and the best linear unbiased predictor (BLUP).”

Minor comments:

2. The bias calculations rely on having a different detection threshold for trait-increasing vs. trait-decreasing minor alleles. This makes sense, but it isn’t clear how asymmetry in the detection thresholds corresponds to asymmetry in the GWAS study design. Do asymmetric detection thresholds only apply to ascertained case-control studies, or can they also arise in an unascertained study with imbalanced case-control ratios? For different case-control ratios, how much do the best-approximation detection thresholds differ, and how much bias does this produce?

We agree that the relationship between unequal detection thresholds in our model and the effects of ascertainment and imbalanced case-control ratios, phenomena described in part by Chan et al. 2014, is not immediate. We ultimately decided that explicit modeling of a case-control study was outside the scope of the present work. However, we believe that the relatively brief discussion of the implications of asymmetric detection thresholds for the bias is still, at least conceptually, useful---particularly in the context of Chan et al. 2014’s findings.

As such, we have emphasized that asymmetric detection thresholds do not precisely correspond to the emergent detection asymmetry observed in Chan et al. 2014. In the introduction, we have added in lines 137-138: “While unequal thresholds do not precisely capture the phenomena described in \cite{Chan2014}, they do...”

In addition, we added the following qualifying sentence to the discussion, lines 661-6663: “Additional work is needed to incorporate these features of case-control studies into our modeling framework.” This further emphasizes that we are not making any strong claims about our model capturing salient features of case-control studies (sample size imbalances, and (epistatic) disease inheritance).

3. From a practical standpoint, a major barrier for cross-population PRS comparisons is population stratification, and perhaps this is also relevant to ancestral population PRS under some demographies. Say the modern population has structure aligned with an ancient admixture event, and the ancestral sample comes from one of the source populations – would uncorrected stratification be expected to produce bias?

We agree that this was not sufficiently commented on in the text. We have now added population stratification to our discussion of the limitations of our model, lines 720-722: "Population stratification in the GWA study population may also lead to biased ancient polygenic scores, as has been observed in cross-population predictions in humans \cite{Berg2019, Sohail2019}."

4. The authors state in the Discussion that observed decay in cross-population prediction accuracy exceeds their theoretical expectations due to AF changes alone, and it might be explained by differences in LD. I think this is a key finding and some quantification is warranted.

Please see our response to Reviewer #1's comment (2). We have now provided a more quantitative comparison between our results and those of Wang et al. 2020 in the Supporting Information S1.12 and reference this in the Discussion.

5. The model assumes that any differences between mean phenotypic values are driven by changes in allele frequency, which is clearly not the case for a trait like height. I'm not bothered by this at all – it's appropriate to focus on genetic components – but some readers probably be, and more discussion might be warranted.

We have addressed other potential factors in the discussion, see our response to reviewer #1's comment (7):

We have added additional discussion of changes in environment and GxE to the final paragraph, lines 714-717: "Indeed, differences in causal effects across contemporary populations, **perhaps due to changes in the environment, epistasis, or gene-by-environment interactions**, likely contribute to accuracy reductions...Indeed, Cox et al..."

6. I thought the contrast between the behavior of the MSE and the additive variance was especially interesting.

Thank you!
