# Supplementary material for: Polygenic score accuracy in ancient samples: Quantifying the effects of allelic turnover
Source: PLoS Genet. 2022 May 6;18(5):e1010170. doi: 10.1371/journal.pgen.1010170 (PMC9116686; doi:10.1371/journal.pgen.1010170)
Supplement: Attachment — Submitted filename: responses_to_reviewers.pdf [file pgen.1010170.s004.pdf]

Response to the second review:

Dear Drs. Lohmueller and Payseur,

Thank you very much for the additional feedback on our manuscript. Below, we have provided a response to the remaining reviewer comments (in blue), including references to the corresponding changes in the manuscript.

The substantive changes in the main text, confined to lines 685-694 in the Discussion, are indicated in red (with line numbers referring to the marked up version of the manuscript). In addition, we have modified Section S1.4.2 of the Supplementary Information.

. . .

Reviews and response to reviews:

Reviewer #1: The authors have addressed my first-round comments sufficiently, and I have no further comments. This was a nice paper even before the revision.

Reviewer #2: The authors have addressed all of my concerns. I recommend this paper for acceptance.

Reviewer #3: In their revised manuscript, Carlson et al. have addressed most of my comments from the initial submission, but I was not completely satisfied by their treatment of the BLUP PRS estimator, which I still think would produce qualitatively different results than the threshold-style PRS estimator they primarily analyze.

The approximation the authors use for the PRS is that there is some detection threshold based on the true effect size (and AF) of a variant, and we get a good estimate of the effect size for variants above the threshold, but a zero estimate for variants below it. This approximation is aligned with a threshold-style estimator, where you throw out variants using a threshold for their estimated (rather than true) effect size/significance.

It contrasts with the BLUP estimator, which does not throw out any variants. The new supplementary note seems to be arguing that the BLUP is approximately the same as the estimator under consideration because its weights are well approximated by the OLS effect size estimates. This is true (perhaps even obvious), but the OLS effect size estimates are only used in the threshold approximation *for variants passing the threshold*, and this threshold seems to be critical reason for the qualitative findings throughout the paper.

As I noted before, the difference between these estimators corresponds to the difference between a sparse vs. infinitesimal genetic architecture: under a sparse architecture, a method with some sort of feature selection is called for; under an infinitesimal architecture, when every SNP is causal, the BLUP estimator is optimal.

I do not at all think it would detract from the findings of the paper if they are only applicable to sparse architectures and threshold/variable selection style estimators. We know that real genetic architectures are not infinitesimal (especially for non-psychiatric traits), and in general, models with some thresholding or variable selection perform better than BLUP.

I don't request new analyses to address this, but I am concerned that the new supplementary note misses the mark, and I recommend at least some qualitative description of what would change under a BLUP-type estimator with no threshold.

Thank you very much for your comments on the revised manuscript. We have now revised the Supplementary Section S1.4.2 to better address the question of how our conclusions would change in the context of a dense trait architecture and a BLUP-type estimator. In particular, we have:

1. Provided expressions for the accuracy and relative accuracy under the assumption that the detection threshold is shared across all sites. In particular, we set the threshold to one such that every segregating site is detected. This formalizes the notion that a BLUP-type estimator can attribute non-zero effects to all sites, regardless of significance.
2. Speculated on the consequences of uncertainty in the effect estimates in a BLUP-type estimator on ancient polygenic score accuracy.
3. Prefaced our (tentative) treatment of BLUP with a discussion of the challenges posed by linkage disequilibrium.

We reference our discussion of the BLUP-type estimator in the Discussion in lines 685-694.

In addition, we have made the following minor changes:

1. In the abstract, we changed "the correlation between an ancient individual's polygenic score and true phenotype" to "expected sample correlation between the polygenic scores of ancient individuals and their true phenotypes" (lines 18-19).
2. Abbreviated linkage disequilibrium in lines 84 and 675.
3. We changed references to sections in the main text to the section titles instead of the section numbers in lines 218, 289-290, and 308.
4. Modified the sentence in lines 598-600 referencing the UK Biobank section to "Consideration of several effect size distributions in a parameter regime consistent with the UK Biobank further supports this conjecture."
5. Removed the word "Indeed" in line 698.
6. All instances of "i.e." are now followed by a comma, i.e., "i.e.,".
