# Supplementary material for: Polygenic score accuracy in ancient samples: Quantifying the effects of allelic turnover
Source: PLoS Genet. 2022 May 6;18(5):e1010170. doi: 10.1371/journal.pgen.1010170 (PMC9116686; doi:10.1371/journal.pgen.1010170)
Supplement: Attachment — Submitted filename: Maryn_PG_PRS_review.docx [file pgen.1010170.s002.docx]

In this manuscript, Maryn et al. provide theoretical results for the effects of mutation and drift on the performance of polygenic scores estimated from a modern population. They focus on the performance of these scores in an ancestral population (from which we might have archaeological samples), and they note that the same calculations are applicable to different contemporaneous populations (at least under a simple demography).

I quite liked the paper. Most of my initial concerns were addressed in the SI. I have one major comment related to the PRS approximation, and some more minor comments more related to presentation.

1. The authors use an approximation for the behavior of a PRS: at some threshold that depends on the sample allele frequency and the true effect size, the effect of that SNP is observed (with no estimation error), and at any lower threshold it is unobserved. This approximation was previously used by Simons et al 2018, and it is clearly a useful one, as they justify in SI.

The authors state that their approximations are insensitive to the true genetic architecture (distribution of beta), but I don’t quite agree. The threshold model is aligned with the “pruning + thresholding” PRS method, and this method can be viewed as an approximate shrinkage/Bayesian posterior approach. In particular it is justified under sparse genetic architectures: only a small proportion of SNPs have a true nonzero effect, so for any SNP that you can’t tell if it’s zero, it is probably zero and you should not put it in the score.

Under an infinitesimal genetic architecture, a totally different approach is called for, using a Gaussian prior. The resulting posterior mean/MAP estimator is variously known as BLUP, ridge regression or LDpred-inf. With this approach, the PRS weights are simply

$$\hat{\beta}=cX^{T}Y$$

for some $c$ depending on the sample size, the sample allele frequency, and the prior effect-size variance (but not depending on $Y$ or $\beta$). I expect that this approach would behave totally differently from the threshold model: every SNP has the same estimation error in per-normalized-genotype units, there is no bias, and the total amount of error depends critically on the number of segregating sites.

I think that state-of-the-art PRS methods can be viewed heuristically as doing something in between BLUP and pruning + thresholding.

To finally state a question, will there be a qualitative difference in the performance of polygenic scores for an extremely polygenic trait coupled with a BLUP estimator?

Minor comments

2. The bias calculations rely on having a different detection threshold for trait-increasing vs. trait-decreasing minor alleles. This makes sense, but it isn’t clear how asymmetry in the detection thresholds corresponds to asymmetry in the GWAS study design. Do asymmetric detection thresholds only apply to ascertained case-control studies, or can they also arise in an unascertained study with imbalanced case-control ratios? For different case-control ratios, how much do the best-approximation detection thresholds differ, and how much bias does this produce?
3. From a practical standpoint, a major barrier for cross-population PRS comparisons is population stratification, and perhaps this is also relevant to ancestral population PRS under some demographies. Say the modern population has structure aligned with an ancient admixture event, and the ancestral sample comes from one of the source populations – would uncorrected stratification be expected to produce bias?
4. The authors state in the Discussion that observed decay in cross-population prediction accuracy exceeds their theoretical expectations due to AF changes alone, and it might be explained by differences in LD. I think this is a key finding and some quantification is warranted.
5. The model assumes that any differences between mean phenotypic values are driven by changes in allele frequency, which is clearly not the case for a trait like height. I’m not bothered by this at all – it’s appropriate to focus on genetic components – but some readers probably be, and more discussion might be warranted.
6. I thought the contrast between the behavior of the MSE and the additive variance was especially interesting.
